# *Trichinella spiralis*-derived extracellular vesicles induce regulatory T cells and reduce airway allergy in mice

**DOI:** 10.3389/fimmu.2025.1637569

**Published:** 2025-07-11

**Authors:** Sofija Glamočlija, Ljiljana Sabljić, Anna Schmid, Nataša Radulović, Alisa Gruden-Movsesijan, Saša Vasilev, Aleksandra Inić-Kanada, Ursula Wiedermann, Irma Schabussova, Maja Kosanović

**Affiliations:** ^1^ Institute for the Application of Nuclear Energy, INEP, University of Belgrade, Belgrade, Serbia; ^2^ Institute of Specific Prophylaxis and Tropical Medicine, Centre for Pathophysiology, Infectiology and Immunology, Medical University of Vienna, Vienna, Austria; ^3^ Institute for Biological Research “Siniša Stanković”, University of Belgrade, Belgrade, Serbia

**Keywords:** *Trichinella spiralis*, extracellular vesicles, respiratory allergy, regulatory T cells, immune modulation, allergic inflammation

## Abstract

**Introduction:**

Respiratory allergies are an increasing global health concern, with current treatments primarily targeting symptoms rather than underlying immune dysregulation. *Trichinella spiralis*-derived extracellular vesicles (TsEVs) have been implicated in modulating immune responses, but their role in allergic airway inflammation remains unexplored. This study investigates the immunomodulatory potential of TsEVs in mitigating ovalbumin (OVA)-induced allergic airway inflammation in mice.

**Methods:**

TsEVs were isolated from *T. spiralis* muscle larvae excretory-secretory products and characterized using nanoparticle tracking analysis. BALB/c mice were sensitized and challenged intranasally with OVA to induce respiratory allergy. TsEVs were administered intranasally before and during OVA challenge. Bronchoalveolar lavage fluid (BALF), lung tissue, spleens, and sera were analyzed for immune cell infiltration, cytokine production, regulatory T cell (Treg) expansion, and OVA-specific antibodies using histology, flow cytometry, and ELISA.

**Results:**

Intranasal administration of TsEVs significantly reduced eosinophilic infiltration and airway inflammation in OVA-sensitized mice. TsEVs treatment suppressed Th2 cytokines (IL-4, IL-5, IL-13) and OVA-specific IgE while enhancing IL-10 production. Importantly, TsEVs promoted expansion of CD4^+^FoxP3^+^ and CD4^+^FoxP3^-^IL-10^+^ regulatory T cells in lungs and spleen, contributing to a systemic anti-inflammatory profile. *Ex vivo* studies confirmed TsEVs-mediated modulation of allergen-stimulated immune responses.

**Discussion:**

Our findings highlight TsEVs as a promising therapeutic approach for allergic airway diseases by promoting immune tolerance and dampening inflammatory responses. These results pave the way for future translational applications of parasite-derived EVs in allergy treatment.

## 1 Introduction

Respiratory allergies have become a major global health problem, affecting 10–30% of the population in urban areas, with prevalence continuing to rise, especially in children (1–3). These allergies have a significant impact on quality of life, causing physical discomfort and emotional distress, as well as social and economic challenges (4, 5).

Respiratory allergies are often triggered by common, otherwise harmless environmental substances that are usually tolerated by the immune system (6, 7). However, disturbance in immune regulatory mechanisms can lead to a failure to maintain homeostasis, resulting in allergic reactions. Respiratory allergies are developed when allergens interact with the nasal epithelium and trigger the release of alarmins that activate innate and adaptive immune responses in the lungs. This cascade increases the levels of type 2 cytokines (IL-4, IL-5, and IL-13), which drive allergen-specific immunoglobulin E (IgE) production through B cell class-switching and recruit eosinophils and neutrophils into inflamed tissues (8–10).

The most common treatments for respiratory allergies involve medications that primarily treat the symptoms without addressing the underlying causes of allergic disease and are often associated with adverse side effects (11–13). Other approaches, such as de-sensitization therapy, provide immunomodulatory benefits but are not effective for all allergens and may not be suitable for every patient (14–16).

Epidemiological studies have shown an intriguing inverse relationship between allergic diseases and parasite infections in regions with higher parasite prevalence (17–19). It was found that parasites can modulate allergic reactions through different mechanisms. Thus, *Echinococcus multilocularis*-derived-extracellular vesicles (EVs) alleviate respiratory allergy airway inflammation in mice by inhibiting macrophage M2a polarization and eliciting Th1 response (20). Other helminths, though infections or their products, have been shown to alleviate allergy symptoms by activating regulatory mechanisms and anti-inflammatory cytokines such as IL-10 and TGF-β (21–26). For example, *Heligmosomoides polygyrus* infection increased levels of IL-10^+^ regulatory B (Breg) and IL-10^+^ Treg cells (21, 23); *Schistosoma mansoni* infection also elicits Treg response in allergic mice (25); recombinant cysteine protease inhibitor of *Ascaris lumbricoides* induce significant increase of regulatory T cells (Tregs) and elevated IL-10 levels (22) while *Oesophagostomum dentatum* extract induce production of regulatory cytokines IL-10 and TGF-beta (26).

*Trichinella spiralis* is characterized by strong immunoregulatory properties that enable modulation of the host’s immune system and thus ensure its long-term survival (27). *T. spiralis* muscle larvae (L1) communicates with the host and exerts its influence through excretory-secretory (ES L1) products, creating an environment dominated by anti-inflammatory and regulatory mechanisms (28, 29). This parasite modulates immune response not only to itself but also to bystander antigens, such as autoantigens or allergens. Indeed, our studies, as well as that from other authors, have shown that infection with *T. spiralis* (30–33) or administration of ES L1 products (29, 34–38) can reduce symptoms of allergy or autoimmune diseases.

Extracellular vesicles (EVs) are particularly interesting components of ES products. EVs are nanosized structures limited by lipid bilayer, released by all cells, crucial for intracellular communication and involved in both physiological (39) and pathological processes (40, 41). They facilitate cross-organism communication, including between parasite and the host (42, 43), and have shown potential for modulating autoimmune and allergic responses (20, 44, 45).

We have shown that *T. spiralis* releases EVs within its ES L1 products (TsEVs) (46). These EVs, isolated by differential centrifugation and ultrafiltration, are 30–80 nm in size (measured by TEM) and carry glycoproteins with immunodominant epitope specific to the muscle larvae of the genus *Trichinella*. We have also revealed that TsEVs possess immunomodulatory properties, as they promote the release of anti-inflammatory cytokines and the development of Treg by affecting human dendritic cells *in vitro* (47). Dendritic cells treated with TsEVs *in vitro* acquire a tolerogenic phenotype characterized by increased expression of tolerogenic markers IDO-1 and ILT3 and elevated production of IL-10 and TGF-β. Others have demonstrated that TsEVs can alleviate TNBS-induced colitis in mice by reducing epithelial barrier damage and significantly decreasing pro-inflammatory cytokines such as IL-1β, TNF-α, IFN-γ, and IL-17A while increasing the percentage of CD4^+^CD25^+^FoxP3^+^ Treg cells in mesenteric lymph nodes (48). Another study on DSS-induced colitis in mice found that treatment with TsEVs reduced expression of proinflammatory cytokines, while at the same time increased expression of cytokines IL-10 and TGF-β in cells from the colonic tissue (49).

Here we aim to investigate the immunomodulatory potential of TsEVs isolated from ES L1 in reducing OVA-induced respiratory allergy, as a proof-of-concept study. The results show that the therapeutic intranasal application of TsEVs impairs the recruitment of eosinophils into the lung, suppresses OVA-specific IgE and Th2 cytokine production, and promotes the expansion of both FoxP3^+^ and FoxP3^-^ Treg cells. These results emphasize the potential of TsEVs as a promising therapeutic option for the treatment of allergic inflammation.

## 2 Materials and methods

### 2.1 Ethics statement

Adult male Wistar rats were used for maintaining the *T. spiralis* strain (ISS 7564) for two months, after which *T. spiralis* muscle larvae (ML) were isolated. Experiments involving OVA-induced respiratory allergy were conducted with female BALB/c mice, aged 6 to 8 weeks. All animals were obtained from the Military Medical Academy (MMA) in Belgrade, Serbia, and housed under standard conditions. The experiments were approved by the Animal Experimentation Committee of the Medical University of Vienna and the Austrian Federal Ministry of Education, Science and Culture (BMBWF-66.009/0277-V/3b/2019), Ethics Committee of the Institute for Application of Nuclear Energy (INEP) and the Ministry of Agriculture, Forestry and Water Management, Republic of Serbia (Permission No. 04–298 and 02-980/1) and were in accordance with EU directive 2010/63/eu on the protection of animals used for scientific purposes.

### 2.2 Preparation of the ES L1 products

*T. spiralis* ML were harvested, cultured and ES L1 products were prepared using established protocols (50, 51). Briefly, fifteen Wistar rats were orally inoculated with 8,500 *T. spiralis* ML/rat, and after two months, ML were collected by artificial digestion. The isolated ML (total number approximately 7.4 x 10^6^, i.e. 0.49 x 10^6^/rat) were washed with sterile phosphate-buffered saline (PBS: 137 mM NaCl, 2.7 mM KCl, 8.1 mM Na_2_HPO_4_, 1.5 mM KH_2_PO_4_) containing 200 μg/ml gentamycin (Carl Roth, Lauterbourg, France), seeded at 5,000 ML per 1 ml in complete Dulbecco’s Modified Eagle Medium (DMEM) (Corning, Mediatech, Manassas, VA, USA) with 200 μg/ml gentamycin (Carl Roth, Lauterbourg, France), and incubated under controlled conditions (37°C, 5% CO_2_) for 18 hours. Total culture supernatant (approximately 1.4 l) was collected, filtered using Fil-tropur V100, 0.22 µm, 1000 ml filter (Sarstedt, Nümbrecht, Germany), and concentrated with an Amicon bioseparation stirred ultrafiltration cell on a 10 kDa membrane (Merck Millipore, Billerica, MA, USA), maintaining sterile conditions throughout. Obtained ES L1 products were then washed with sterile PBS and concentrated using an Amicon bioseparation stirred ultrafiltration cell on a 10 kDa membrane. The protein concentration in the ES L1 products (694 µg/ml) was determined using the Pierce BCA protein assay kit (Thermo Fisher Scientific, Dreieich, Germany) according to the manufacturer’s instructions.

### 2.3 Enrichment of EVs

EVs from *T. spiralis* muscle larvae (TsEVs) were enriched following the methods outlined in previous research (46). The enrichment process involved differential centrifugation followed by ultrafiltration, keeping sterile conditions. ES L1 products (starting volume 22 ml), filtered through a 0.22 μm filter, were submitted to differential centrifugation, first at 300 x g for 10 minutes at 4°C, followed by 20 minutes at 3,000 x g. This was succeeded by ultracentrifugation at 17,000 x g for 25 minutes and then at 110,000 x g for 2 hours (k-factor = 307.4) using an SW 41 Ti rotor in an Optima L-90K ultracentrifuge (Beckman Coulter, Indianapolis, IN, USA). After each centrifugation step, the supernatant was collected for further enrichment. The final pellet obtained after centrifugation at 110,000 x g was resuspended in 100 µl of sterile PBS. Ultrafiltration was utilized to remove soluble proteins smaller than 100 kDa, during which the total volume of TsEVs was washed with six volumes of sterile PBS using a Vivaspin 500 membrane (100 kDa MWC0 PES; Sartorius Stedim Lab Ltd, Stonehouse, UK). More extensive characterizations of TsEVs were published earlier (46, 47).

### 2.4 Endotoxin detection

Endotoxin levels in ES L1 products were measured by the Limulus Amoebocyte Lysate (LAL) turbidimetric assays and were found to be below 0.5 EU/ml, which is within the acceptable limit established by the U.S. Food and Drug Administration. Following testing, the products were sterile-filtered through a 0.22 μm filter (Sarstedt, Nümbrecht, Germany) and either used immediately for TsEVs enrichment or stored at -20°C until further use. Enriched TsEVs were tested for the presence of LPS using HEK-Blue-mTLR4 cells carrying a SEAP reporter construct (InvivoGen, San Diego, CA, USA). Results were found to be at the level of negative control (blank) and are presented in the **Supplementary Figure S1**.

### 2.5 Nanoparticle tracking analysis

**Figure 1:**
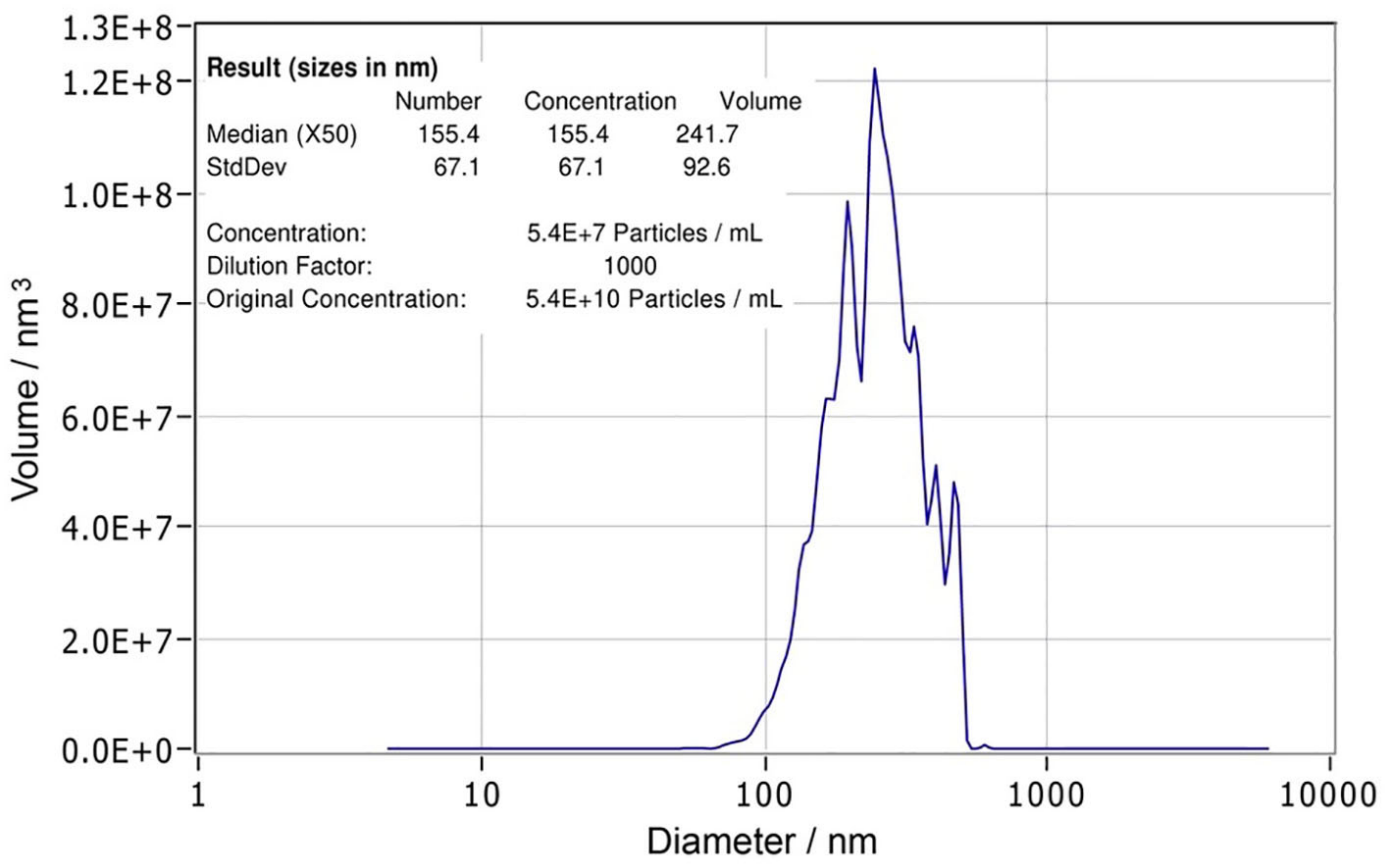
Characterization of *Trichinella spiralis* extracellular vesicles. Nanoparticle tracking analysis (NTA) report of *T. spiralis* extracellular vesicles with statistical analysis indicating the size (155.4 ± 67.1 nm) and the concentration (5.4 x 10^10^ (p/ml)) of the vesicles.

Nanoparticle tracking analysis (NTA) is a widely utilized technique for simultaneously assessing both the size distribution and concentration of particles. NTA was conducted using the ZetaView PMX-420 QUATT nanoparticle tracking instrument (Particle Metrix, Inning am Ammersee, Germany) in scatter mode. The TsEVs sample was diluted to 1:1000 PBS for analysis, and measurements were taken at 11 positions with the following settings: maximum area 1000, minimum area 10, minimum brightness 20, sensitivity 78, and shutter 100. Particle size and concentration were analyzed using ZetaView software version 8.05.16 SP3 (Particle Metrix, Inning am Ammersee, Germany). Final particle concentration was determined to be 10^10^ particles/ml (p/ml) and TsEVs was aliquoted at 10^8^ particles per 10 μl and stored at -80°C until further use (Data on isolated TsEVs are presented in **Figure 1**).

### 2.6 Experimental design

**Figure 2:**
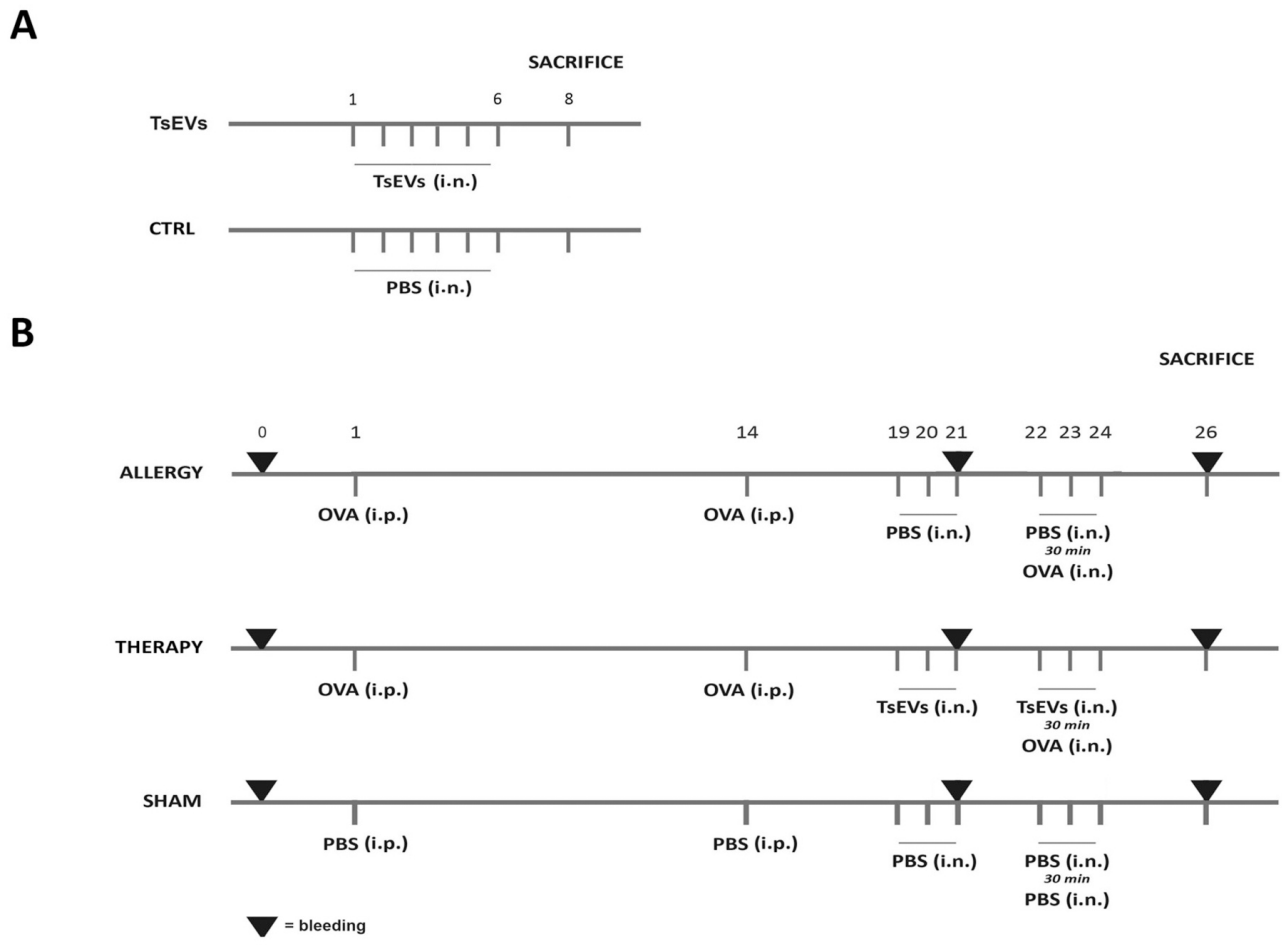
Experimental design. **(A)** TsEVs treatment in healthy mice: Mice received either 0.5 x 10^8^ TsEVs (TsEVs group) or PBS alone (CTRL group) via intranasal (i.n.) administration for six consecutive days. **(B)** Mice were sensitized with intraperitoneal (i.p.) injections of ovalbumin (OVA, 100 μg) on days 1 and 14 and challenged with OVA via i.n. administration on days 22 to 24 (Allergy group). Control group received PBS instead of OVA (Sham group). Mice followed the same protocol as in Allergy group and were treated i.n. with TsEVs (0.5 x 10^8^) TsEVs on days 19 to 21 and 30 min before every challenge on days 22 to 24.

Three groups of BALB/c mice were used (n=5) in experiments testing TsEVs potential to modulate allergic airway inflammation (**Figure 2B**). In Allergy group, airway inflammation was induced by intraperitoneal (i.p.) injection of 10 μg OVA (Sigma–Aldrich, Darmstadt, Germany) and 67% (v/v) alum (Alu-Gel-S Suspension; Serva Electrophoresis, Heidelberg, Germany) suspension (total volume of 150 μl), on days 1 and 14 (sensitization days), followed by intranasal (i.n.) challenge from days 22 to 24 with 100 μg of OVA in 30 μl of PBS per mouse for each administration (challenge days) according to Korb et al. (52). The control group (Sham) received PBS and alum suspension i.p. on days 1 and 14, and PBS i.n. from days 19 to 24. In the Therapy group, allergic airway inflammation was induced as aforementioned, and mice were treated i.n. with 0.5 x 10^8^ TsEVs/30 μl (16.7 × 10^8^ TsEVs/ml per mouse) on days 19 to 21 and on challenge days (from day 22 to 24), 30 minutes before i.n. application of OVA. The total amount of TsEVs administered per mouse, during six consecutive days, was 3 x 10^8^/180 µl.

To assess the effects of TsEVs in the absence of airway inflammation on healthy animals (**Figure 2A**), mice were administered with 0.5 x 10^8^ TsEVs/30 μl i.n for 6 consecutive days, receiving the same amount of TsEVs as used for the treatment of respiratory allergy (TsEVs group). The control group for this assessment were mice that received i.n. PBS (Ctrl group).

Prior to each i.n. administration, mice were anesthetized with 5% (v/v) isoflurane (Vetflurane, gaseous anesthetic, Virbac) at an airflow rate of 3 L/min using a R650-IE Veterinary Anesthesia Machine (RWD, Guangdong, China). Blood was collected on day 0, day 21 (before challenge with OVA) and on day 26 as explained in section 2.12. Mice from each experimental and control group were sacrificed at day 26, and lungs and spleens were aseptically removed for further examination.

### 2.7 Characterization of cell populations in the bronchoalveolar lavage fluid

Isolation and characterization of BALF cells was performed according to Korb et al. (52). To identify cell populations present in BALF, the lungs were lavaged twice with 500 µl of ice-cold PBS. The collected fluid was then centrifuged (300 x g for 5 minutes at 4°C), and pelleted cells were resuspended in PBS from which a total of 5 x 10^4^ cells were spun onto microscope slides (800 x g for 5 minutes), air-dried, and stained with hematoxylin and eosin (H&E). Slides were scanned using the Tissue FAXSi Plus system, and cells were counted in the Tissue FAXS viewer (TissueGnostics, Vienna, Austria) with at least 150 cells, including macrophages, eosinophils, lymphocytes, and neutrophils counted per slide.

### 2.8 Lung histology

Histological evaluation of the lungs was carried out following a published protocol (52). The lungs were fixed in 7.5% formaldehyde (SAV Liquid production, Flintsbach, Germany). Formaldehyde-fixed lungs were dehydrated with a series of ethanol solutions, followed by xylene, and subsequently embedded in paraffin. Tissues were sectioned at a thickness of 3 µm, capturing four different tissue depths separated mutually ~60 μm. At least two slides, each containing four different cross-sections, for each animal, were stained with either H&E or Periodic acid-Schiff (PAS). The histological pathology score was evaluated according to Zaiss et al. (53) with modifications. Sections were semi-quantitatively scored by analyzing five different regions on each sample, for the H&E staining using the following criteria: analysis of inflammatory cells in peribronchial and perivascular areas as 0 = no inflammation; 1 = few inflammatory cells; 2 = layer of 1–4 inflammatory cells; 3 = layer of 4–10 inflammatory cells; 4 = layer of more than 10 inflammatory cells; while for the PAS staining, mucus secretion, presence and hyperplasia of goblet cells was scored as 0 = no goblet cells; 1 = mild mucus secretion, 1/3 of epithelium occupied by goblet cells; 2 = moderate mucus secretion, 1/3–2/3 of epithelium occupied by goblet cells; 3 = high mucus secretion and > 2/3 of epithelium occupied by goblet cells.

### 2.9 Lung cell isolation and restimulation *ex vivo*

Isolation and cultivation of lung cells was performed as described previously (52). Lungs of terminally anesthetized mice were minced and incubated in 2 ml RPMI 1640 (Biowest, Nualle, France) with 100 μg/ml gentamycin (Carl Roth, Lauterbourg, France) and containing 50 µg/ml Liberase TL (Roche, Mannheim, Germany) and 500 ug/ml DNAse (Sigma-Aldrich) for 1 hour at 37°C in 5% CO_2_ atmosphere. Next, the digested tissue was forced through a 70 μm cell strainer and erythrocytes were lysed in 3 ml of lysis buffer (155 mM NH4Cl, 12 mM NaHCO3, 0.1 mM EDTA in ddH20) for 5 minutes. Cell number and viability was assessed using Muse Count and Viability Kit on the Luminex Guava Muse Cell Analyzer (Luminex, Austin, Texas, USA). Cells were resuspended in RPMI 1640 containing 10% Fetal Calf Serum (FCS) (Gibco, Thermo Fisher Scientific) and 100 μg/ml gentamycin (all Sigma-Aldrich) after which cells intended for flow cytometry analysis (4 x 10^6^/ml) were stimulated with phorbol-12-myristate-13-acetate (PMA) 20 ng/ml, calcium ionophore (Ca-I) 500 ng/ml and monensin (2 μM) (all from Sigma-Aldrich) for 4 hours on 37°C 5% CO_2_ atmosphere.

Cells intended for measurement of cytokine release were resuspended in RPMI 1640 containing 10% FCS, 100 μg/ml gentamycin, 2 mM mercaptoethanol and 2 mM L gluta-mine (both Sigma-Aldrich) were plated at a concentration 5 x 10^6^/ml in 96-well round-bottom plates (Sarstedt, Numbrecht, Germany). Cells were either incubated with medium or were additionally stimulated with OVA 100 μg/ml (Endo- Grade; Hyglos, Bernried am Starnberger See, Germany) at 37°C 5% CO_2_. Additional lung cells from the Allergy group were seeded and restimulated with 100 μg/ml of OVA for 1 hour, after which they were treated with 3 x 10^7^ p/ml of TsEVs, a concentration shown to be effective in our previous study (47). After 72 hours, all cell culture supernatants were collected, and cytokine levels were analyzed using ELISA.

### 2.10 Spleen cell isolation and restimulation *ex vivo*

Isolation and cultivation of spleen cells was performed according to previously published study (52). Spleens of terminally anesthetized mice were collected and pressed through a metal net, after which the disrupted tissue was forced through a 70 μm cell strainer using RPMI 1640 medium with 10% FCS. Mononuclear cells were isolated using Hystopaque-1077 (Sigma–Aldrich, Darmstadt, Germany) density gradient centrifugation, the cells were washed with RPMI 1640/10% FCS, and erythrocytes were eliminated using a lysis buffer. Cell number and viability was assessed using Muse Count and Viability Kit on the Luminex Guava Muse Cell Analyzer (Luminex, Austin, Texas, USA). Similar to lung cells, spleen cells intended for flow cytometric analysis were resuspended (4 x 10^6^ cells/ml) in RPMI 1640 with 10% FBS and 100 μg/ml gentamycin and stimulated with PMA, Ca-I, and monensin for 4 hours.

Spleen cells intended for cytokine release analysis were resuspended in RPMI 1640 containing 10% FCS, 100 μg/ml gentamycin, 2 mM mercaptoethanol and 2 mM L glutamine and were plated at a concentration 5 x 10^6^/ml in 96-well round-bottom plates and additionally stimulated with OVA. Additional spleen cells from the Allergy group were seeded and restimulated with 100 μg/ml of OVA for 1 hour, after which they were treated with 3 x 10^7^ particles/ml of TsEVs. All spleen cell supernatants were collected after 72 hours, and cytokine levels were analyzed with ELISA.

### 2.11 ELISA

To determine cytokine levels of IFN-γ, IL-4, IL-5, IL-10, and IL-13 in lung and spleen cell supernatants, eBioscience Ready-SETGo! ELISA kits (eBioscience, San Diego, CA) were used according to the manufacturer’s instructions. Briefly, flat bottom 96-well-plate (Nunc MaxiSorp, Thermo Scientific, Waltham, MA) were coated with designated capture anti-bodies and incubated at 4°C overnight. The next day, plates were washed with PBS containing 0.05% Tween 20 (Carl Roth, Karlsruhe, Germany) (PBST) and blocked with PBST containing 5% bovine serum albumin (BSA) (Sigma-Aldrich) at room temperature (RT) for 2 hours. Samples and standards were diluted 1% BSA/PBST and incubated at RT for 3 hours. Technical duplicates of the samples were used in all essays. This and all subsequent steps were followed by a washing step with PBST. Biotinylated detection antibodies diluted in 1% BSA/PBST were incubated at RT for 1 hour after which horseradish-peroxidase (HRP) conjugated avidin was added and incubated at RT for 30 minutes. For detection, the TMB substrate was added, and plates were incubated for 15 minutes at RT in the dark. The reaction was stopped using 0.18 M H2SO4, and the absorbance was measured at 450/570 nm in a TECAN Spark 10M plate reader (Tecan Trading AG, Männedorf, Switzerland).

### 2.12 Detection of OVA specific antibodies

Blood samples were collected at the beginning of the experiment (day 0), as well as on days 21 and 26 (sacrifice day). Approximately 100 μl of blood was obtained by puncturing the facial vein. The serum was separated by centrifuging the blood in Microtainer SST tubes (BD Biosciences, San Jose, CA, USA) at 10,000 x g for 5 minutes and stored at −20°C until analysis of OVA-specific IgE, IgA, IgG1, and IgG2a antibodies. Nunc MaxiSorp flat-bottom 96 well plates (Thermo-Fisher Scientific) were coated with 5 μg/ml OVA at 4°C overnight. The next day, plates were washed with PBST and blocked with 1% BSA/PBST for 2 hours at 37°C. After discarding the blocking solution, the serum samples were diluted according to specific antibodies (for detection of IgE antibodies, serums were diluted 1:10, for IgA 1:10, for IgG1 1:2000 and IgG2a 1:500) with 0.5% BSA/PBST and incubated for 2 hours at RT. Serum from mice with confirmed respiratory allergy, verified by flow cytometry and histological analysis, was used as the positive control, while serum from sham-treated (non-sensitized) mice served as the negative control. After incubation the plates were washed with PBST and respective purified rat anti-mouse antibodies were added: anti-IgE (R35-72), anti-IgA (C10-1), anti-IgG1 (A85-1), anti-IgG2a (R19-15) (all BD Biosciences, San Jose, CA, USA); all antibodies were diluted 1:500 with 0.5% BSA/PBST and incubated for 2 hours at RT. After washing, plates were incubated with HRP-conjugated mouse anti-rat IgG antibody (Jackson ImmunoResearch Laboratories Inc., Ely, UK), which was diluted 1:2000 with 0.5% BSA/PBST and incubated for 2 hours at RT. After incubation, the washing step was repeated, and TMB was used for detection while the reaction was stopped with 0.18 M H2SO4. The optical density (O.D.) of antibody levels was measured at 405 nm in a TECAN Spark 10M plate reader.

### 2.13 Flow cytometry

Single-cell suspensions from the lung and spleen were washed once with PBS containing 0.01% NaN3, then blocked for 15 minutes with 2% normal mouse serum to inhibit Fc receptor binding, and subsequently labeled with primary antibodies. Cells were stained with surface markers for 30 minutes at +4°C with the following monoclonal antibodies (mAbs) at dilutions recommended by the manufacturer: anti-Ly6G-Alexa Fluor 488(AF488) (RB-68C5) (Invitrogen Thermo Fisher Dreieich, Germany), an-ti-CD3-Fluorescein Isothiocyanate (FITC) (17A2) BioLegend, Basel, Switzerland, an-ti-CD4-FITC (GK1.5) (Sony Biotechnology, San Jose, CA, USA), anti-MHC class II (I-A)-Phycoerythrin (PE) (NIMR-4) (eBioscience, San Diego, CA, USA), anti-CD19-PE (QA17A27), anti-CD45-PE-Dazzle 594 (30-F11), anti-Ly6C-Peridinin Chlorophyll Pro-tein/Cyanine5.5 (PerCP/Cy5.5) (all BioLegend), anti-CD8a-Peridinin Cyanine 5 (PerCy5) (53-6.7), anti-CD11b-Peridinin Cyanine 7 (PerCy7) (M1/70) (both Invitrogen), anti-TCRβ-PerCy7 (H57-597), anti-CD103-Allophycocyanin (APC)(2E7) (both BioLegend), anti-CD49b-APC (DX5) (MACS Miltenyi Biotec, Cologne, Germany), anti-CD107 (Sig-lecF)-Alexa Fluor 700 (AF700) (1RNM44N) (Invitrogen), anti-CD11c-Allophycocyanin/Cyanine7 (APC-Cy7) (N418) (BioLegend). Intracellular staining of cells was conducted after the surface staining, by using the flow cytometry fixation and permeabilization kit (R&D Systems) according to recommendations. The intracellular staining was carried using following mAbs: anti-IL-17-PE (eBio17B7), anti-IL-4-Biotin (BVD6-24G2), anti-IL-10-Biotin (JES5-2A5) (all eBioscience), anti-LAP (TGF-β)-PE (TW7-16B4), anti-IFN-γ-APC (XMG1.2) (both BioLegend), anti-Foxp3-PE-Cy5 (376209) (R&D systems Minneapolis, MN, USA). Cells stained with biotinylated antibodies were additionally incubated with APC-Cy Streptavidin. Single labeled samples were used for compensation of signal overlap between the channels before each analysis while isotype-matched control mAbs were used to determine non-specific background staining: immunoglobulin (Ig)G1 control-FITC (P3.6.2.8.1), IgG1 control-PE (P3.6.2.8.1), IgG1κ con-trol-PerCP (MOPC-31C), IgG1 control-PECy7 (P3.6.2.8.1), IgG1 control-APC (P3.6.2.8.1) (all eBioscience, San Diego, CA, USA), IgG1κ control-PE/Dazzle (MOPC-21), IgG1κ con-trol-AF700 (MOPC-21) (both BioLegend, Basel, Switzerland) and IgG1 control-APCCy7 (MOPC-21) (Abcam, Cambridge, UK). For each analysis, at least 100,000 cells were acquired. Cells were gated according to their specific side-scatter (SSC)/forward-scatter (FSC) properties on the GloCell Fixable Viability Dye- negative population (viable cells; STEM-CELL technologies, Vancouver, Canada), thereby excluding GloCell-stained dead cells. A minimum of 80,000 CD45^+^ cells were acquired per sample. Labeled cells were analyzed on an 8-colors BD FACS LSR II flow cytometer and collected data was further assessed online using software FlowJo VX (BD Biosciences, San Jose, CA, USA).

### 2.14 Statistical analysis

Results are presented as mean value ± SD from three independent experiments. Statistical analysis was performed in GraphPad Prism 8.0.1 for Windows (GraphPad Soft-ware, San Diego, CA, USA). All data was tested for outliers. The Shapiro-Wilk test was used to determine the distribution of data (data normality), followed by parametric or non-parametric tests. One-way ANOVA with Tukey’s multiple comparisons test or Kruskal-Wallis tests were used to compare the percentage or total number of immune cells within the tested groups. The relative distribution of immune cells was shown as cell percentages calculated gated within differential cell subsets, all of which were gated to total CD45^+^ gated cells. Results from cytokine and OVA-specific antibody ELISA assays were calculated using Two-way ANOVA with Tuckey’s multiple comparison test. In contrast, comparisons between OVA-stimulated and OVA+TsEVs treated cells were done using the Student T-test. Significant differences are marked as *p < 0.05, **p < 0.01, ***p < 0.005, ****p < 0.0001.

## 3 Results

### 3.1 Characterization of TsEVs

TsEVs enriched from ES L1 antigen were analyzed by NTA, which revealed a concentration of 5.4 x 10^10^ particles/ml (corresponding to a total number 5.4 x 10^9^ particles/100 µl). The average size was 155.4 ± 67.1 nm (**Figure 1**).

### 3.2 Intranasal administration of TsEVs enhances CD4^+^Foxp3^+^ and CD4^+^Foxp3^-^IL-10^+^ Treg cells in the lungs of healthy mice

**Figure 3:**
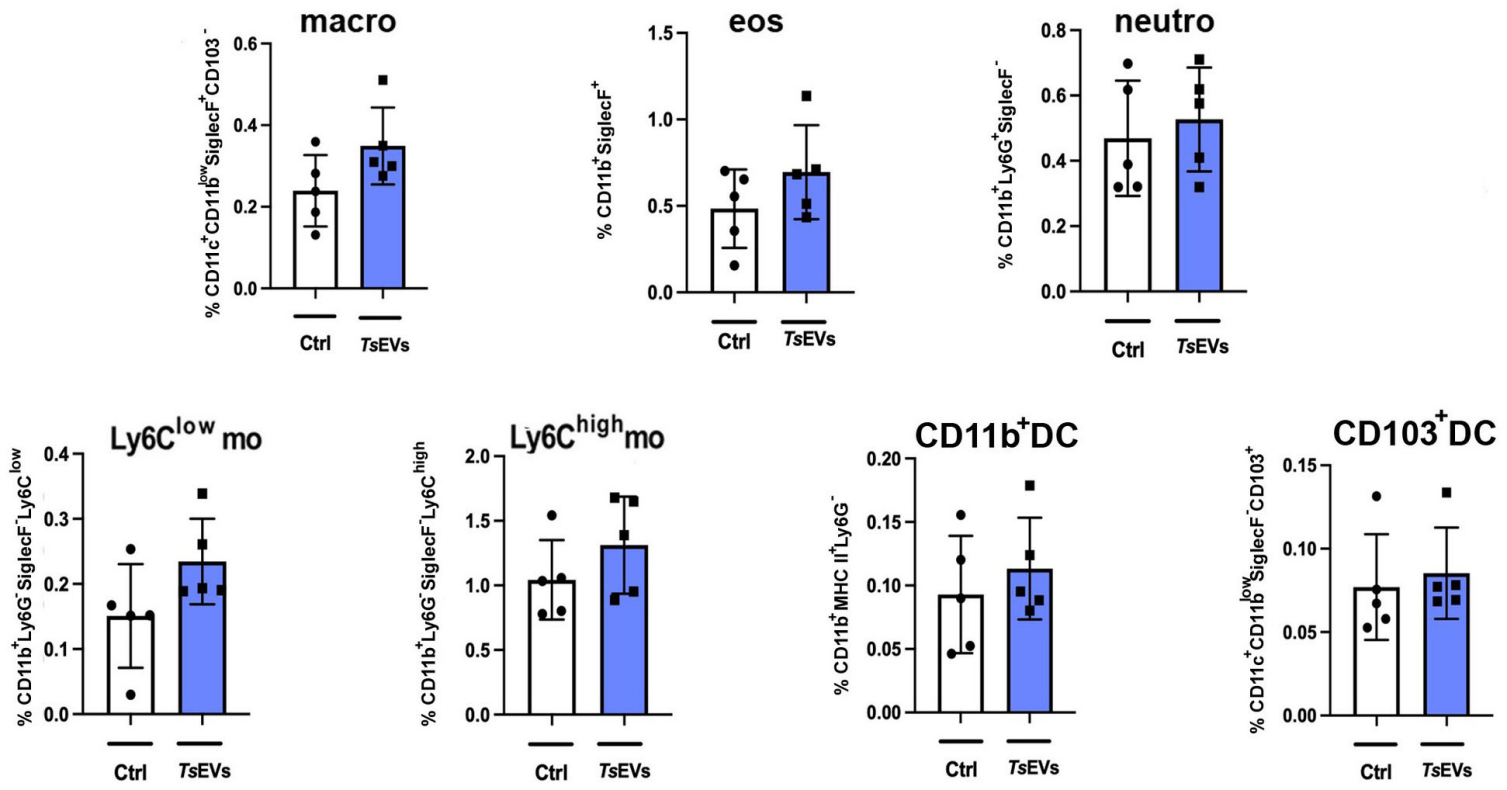
Analysis of myeloid cell populations in the lungs of mice treated with TsEVs. Mice were treated as outlined in **Figure 2A**: animals received either 0.5 × 10^8^/30μl TsEVs or equal volume of PBS (Ctrl) via intranasal administration daily for six consecutive days. Lung tissue was collected on day 8 and processed for flow cytometric analysis of immune cell populations. Bar graphs display both the percentage of myeloid cell subsets present in the lung. These include macrophages (macro), eosinophils (eos), neutrophils (neutro), Ly6C^low^ and Ly6C^high^ monocytes (mo), CD11b^+^ dendritic cells (CD11b^+^ DC), and CD103^+^ DC. Cell subset identification was based on established gating strategies shown in **Supplementary Figure S1**. Statistical comparisons were performed using Student’s t-test.

**Figure 4:**
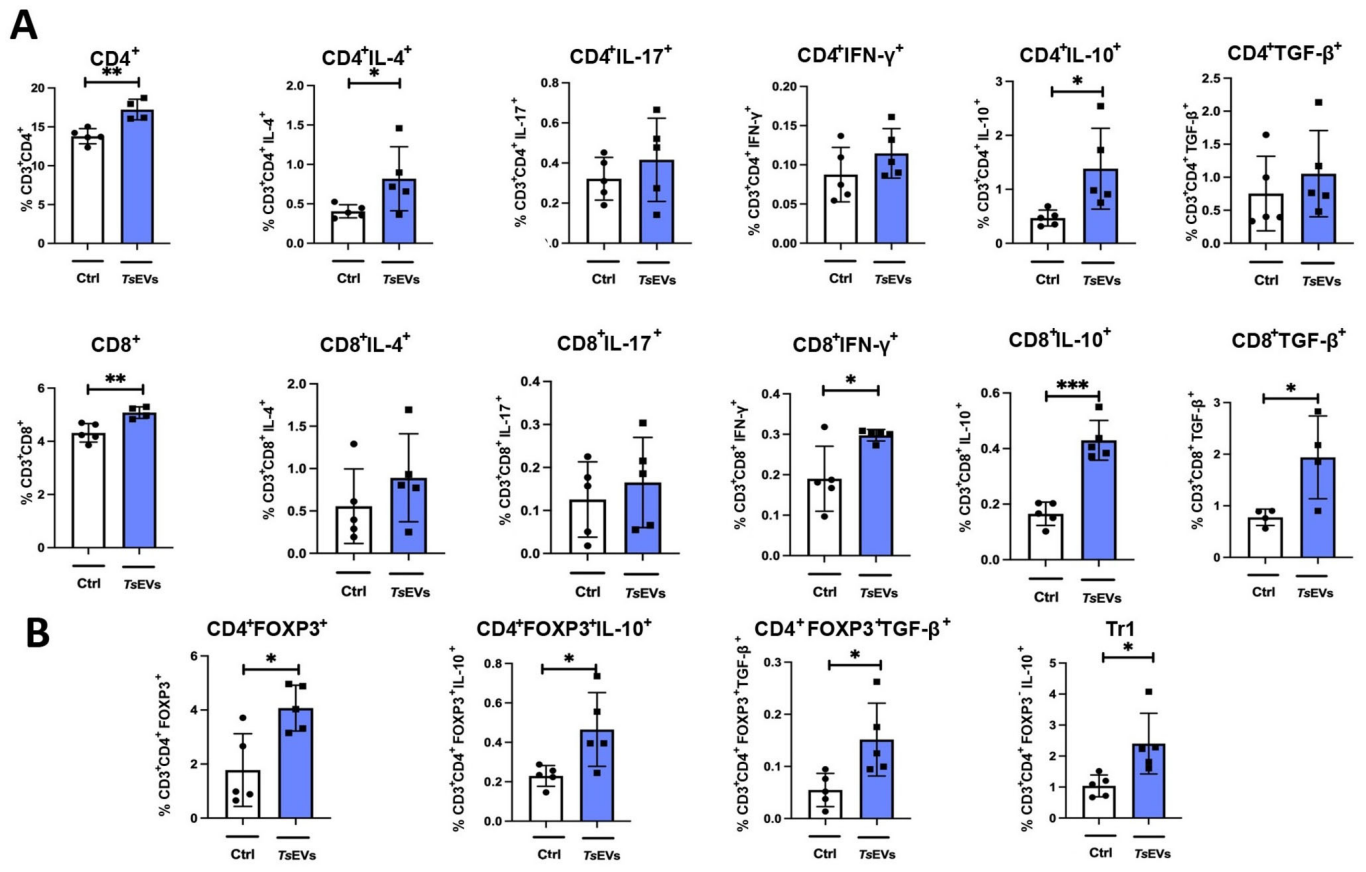
Analysis of lymphoid cell populations in the lung of mice treated with TsEVs. Mice were treated as outlined in **Figure 2A**: animals received either 0.5 × 10^8^/30 μl TsEVs or equal volume of PBS (ctrl) via intranasal administration daily for six consecutive days. Lung tissue was collected on day 8 and processed for flow cytometry to assess immune cell populations. Bar graphs display both the percentage of lymphoid cell subsets present in the lung. **(A)** Proportion of CD4^+^ T cells and CD8^+^ T cells, including subsets positive for intracellular staining of IL-4, IL-17, IFN-γ, IL-10, and TGF-β (see **Supplementary Figure S2** for gating strategy). **(B)** Proportion of CD4^+^Foxp3^+^ Tregs, CD4^+^Foxp3^-^IL-10^+^ (Tr1) cells, and expression of IL-10 and TGF-β within CD4^+^Foxp3^+^ Treg cells (gating strategy shown in **Supplementary Figure S3**). Statistical analysis was performed using Student’s t-test and statistical significance indicated as *p < 0.05, **p < 0.01, ***p < 0.005.

To investigate the effect of TsEVs on the lungs of healthy BALB/c mice, we analyzed immune cell populations involved in innate and adaptive responses following i.n. application of TsEVs (TsEVs group) (**Figures 3**, **4**). A control group received PBS under identical conditions (Ctrl group).

Analysis of myeloid cell subsets in the lung showed no significant differences in the proportion and the total number of alveolar macrophages, eosinophils, neutrophils, monocytes, or DCs following TsEVs treatment (**Figure 3**; **Supplementary Figures S2**, **S5A**). However, examination of lymphoid cell subsets revealed an increased percentage and the total number of both CD4^+^ and CD8^+^ T cells in TsEVs-treated mice (**Figure 4A**; **Supplementary Figures S3**, **S5B**). Cytokine expression analysis further demonstrated a significant increase in the percentage of CD4^+^IL-4^+^, as well as in the percentage and the total number of CD4^+^IL-10^+^cells, CD8^+^IFN-γ^+^, CD8^+^IL-10^+^ and CD8^+^TGF-β^+^ T cells.

The lungs of TsEVs-treated mice exhibited a significant increase in the percentage and the total number of Treg cells, including both CD4^+^Foxp3^+^ Tregs and CD4^+^Foxp3^-^IL-10^+^ Tr1 cells, compared to PBS-treated control (**Figure 4B**; **Supplementary Figures S4**, **S5C**). Furthermore, Tregs in TsEVs-treated mice showed a significant increase in intracellular expression of IL-10 and TGF-β.

### 3.3 Intranasal administration of TsEVs enhances CD4^+^Foxp3^+^ and CD4^+^Foxp3^-^ regulatory cell proportions in the spleens of healthy mice

**Figure 5:**
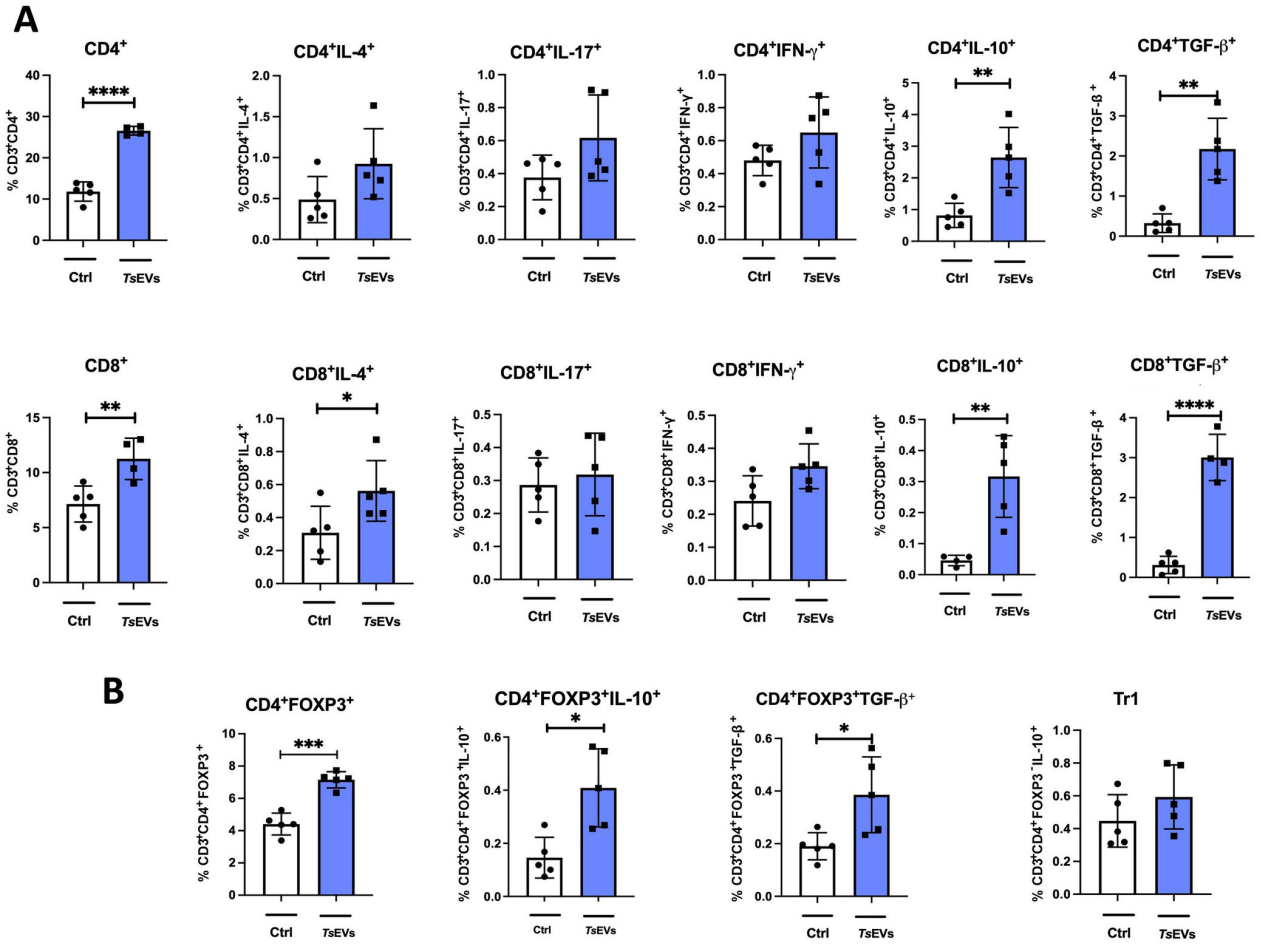
Analysis of lymphocyte populations in the spleen of mice treated with TsEVs. Mice were treated as shown in the **Figure 2A**. Spleen cells were collected and analyzed using flow cytometry. **(A)** Proportion of CD4^+^ T cells, CD8^+^ T cells, and IL-4, IL-17, IFN-γ, IL-10, TGF-β producing CD4^+^ and CD8^+^ T cells in spleen analyzed by flow cytometry (see **Supplementary Figure S2** for gating strategy). **(B)** Proportion of regulatory T cell populations: Treg (CD4^+^Foxp3^+^) and Tr1 (CD4^+^Foxp3^-^IL-10^+^) cells and IL-10 and TGF-β producing Treg cells are presented (see **Supplementary Figure S3** for gating strategy). *p < 0.05, **p < 0.01, ***p < 0.005, ****p < 0.0001 (Student´s t-test).

The systemic effects of intranasal TsEVs administration on the immune system were evaluated by analyzing lymphocyte populations and their cytokine expression in the spleens of treated mice (**Figure 5**). TsEVs treatment significantly increased the proportion and the total number of CD4^+^ T cells in the spleens of TsEVs-treated mice compared to the control (**Figure 5A**; **Supplementary Figures S3**, **S6A**). While the proportion and the total number of CD4^+^ T cells expressing IL-4, IL-17, and IFN-γ remained unchanged, the proportion and the total number of CD4^+^ T cells producing cytokines IL-10 and TGF-β increased significantly.

TsEVs administration also resulted in an overall increase in the percentage and the total number of CD8^+^ T cells in the spleens compared to control mice (**Figure 5A**). Within this expanded population, a significant increase in the percentage of CD8^+^IL-4^+^ cells was observed. However, the proportion and the total number of CD8^+^ T cells expressing proinflammatory cytokines such as IL-17 and IFN-γ remained unchanged. The proportion and the total number of CD8^+^ T cells expressing IL-10 and TGF-β increased significantly, indicating an enhanced anti-inflammatory and regulatory profile within the CD8+ T cell subset (**Figure 5A**).

TsEVs treatment increased the proportion and the total number of CD4^+^Foxp3^+^ T cells in the spleens of treated mice compared to the control (**Figure 5B**; **Supplementary Figures S4**, **S6B**). Within this population, there was a significant increase in the production of IL-10 and TGF-β. In contrast, the treatment with TsEVs did not affect the proportion of Tr1 cells (**Figure 5B**).

Overall, these results highlight the ability of TsEVs to promote a systemic regulatory immune profile, characterized by increased proportions and total numbers of CD4^+^Foxp3^+^ Tregs and CD4^+^ and CD8^+^ T cells producing anti-inflammatory and regulatory cytokines.

### 3.4 Intranasal administration of TsEVs protects against OVA-induced allergic airway inflammation

To investigate the anti-inflammatory potential of TsEVs as a therapeutic agent for respiratory allergies, mice sensitized and challenged with OVA to induce allergic airway inflammation were treated with TsEVs. The treatment was administered i.n. on days 19 to 21 and 30 minutes prior to each OVA challenge on days 22 to 24 (**Figure 2B**).

**Figure 6:**
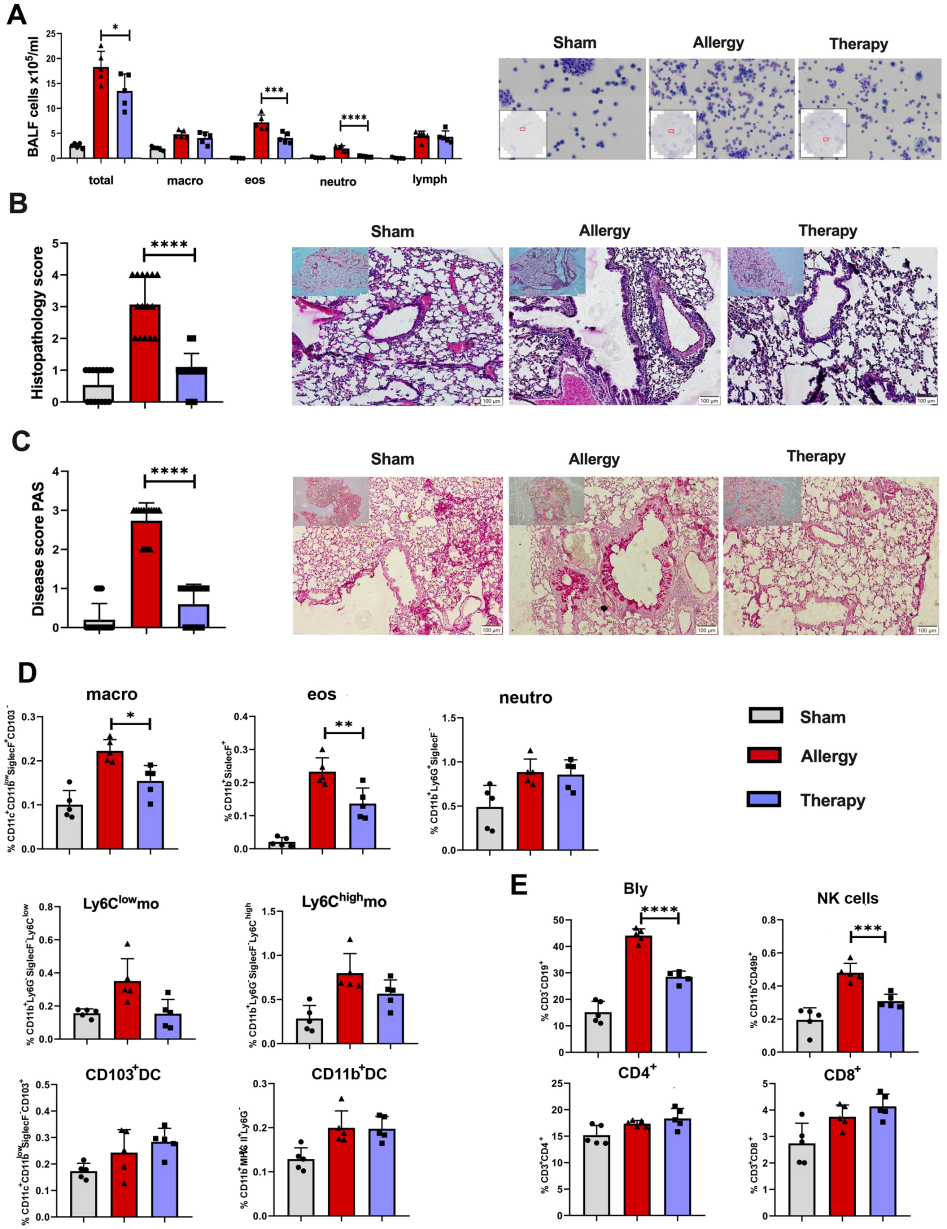
Effects of TsEVs treatment in the lung of mice with allergic inflammation. Mice were treated according to the protocol outlined in **Figure 2B**. Animals were sensitized with intraperitoneal (i.p.) injections of ovalbumin and alum (OVA, 100 μg) on days 1 and 14 and challenged with intranasal (i.n.) application of OVA on days 22–24 (Allergy). The control group (Sham) received PBS instead of OVA. The treatment group received TsEVs (0.5 × 10^8^) via i.n. administration on days 19–21, and 30 minutes prior to each OVA challenge (days 22–24). On day 26, bronchoalveolar lavage fluid (BALF) was collected and lungs were processed for histological and flow cytometric analyses. **(A)** Representative images and differential cell counts of BALF samples from each group. **(B, C)** Representative lung tissue sections stained with hematoxylin and eosin (H&E) and periodic acid–Schiff (PAS), showing peribronchial inflammatory cell infiltration **(B)** and mucus production **(C)**, respectively. Histopathological changes were quantified using disease scoring systems (H&E: scores 1–4; PAS: scores 0–3). Scale bar = 100 µm. **(D)** Bar graphs display the percentage of myeloid cell subsets present in the lung: macrophages (macro), eosinophils (eos), neutrophils (neutro), Ly6C^low^ and Ly6C^high^ monocytes (mo), CD103^+^ dendritic cells (DC) and CD11b^+^ DC (see **Supplementary Figure S1** for gating strategy). **(E)** The proportion of lymphoid cell subsets in the lung: B lymphocytes (Bly), natural killer (NK) cells, CD4^+^ and CD8^+^ T cells (see **Supplementary Figure S2** for gating strategy). Results are presented as mean ± SD of three independent experiments. Statistical analysis was performed using one-way ANOVA with Tukey’s posttest and statistical significance indicated as *p < 0.05, **p < 0.01, ***p < 0.005, ****p < 0.0001 when compared with the Allergy group.

Analysis of BALF revealed a significant reduction in the total cell counts in the Therapy group compared to allergic controls (**Figure 6A**). Specifically, the numbers of eosinophils and neutrophils were significantly reduced, while the proportion of macrophages and lymphocytes remained unaffected by TsEVs treatment.

Histological examination of lung tissue showed severe inflammatory cell infiltrations in the peribronchial and perivascular regions, along with mucus overproduction and goblet cell hyperplasia in allergic mice (Allergy group), as observed in H&E and PAS-stained sections (**Figures 6B, C**). TsEVs treatment (Therapy group) reduced air-way epithelial thickening, decreased inflammatory cell infiltration, and lessened mucus overproduction (**Figures 6B, C**). Semi-quantitative analysis of histological scores confirmed that TsEVs therapy ameliorated these pathological changes.

Flow cytometric analysis of lung myeloid cells showed a significant reduction in the proportion and the total number of alveolar macrophages and eosinophils in the TsEVs-treated group compared to allergic controls (**Figure 6D**; **Supplementary Figures S2**, **S7A**), while other examined myeloid cells, neutrophils, Ly6C^low^ (non-classical) monocytes, Ly6C^high^ monocytes, CD103^+^DC, and CD11b^+^DC were not affected by the treatment.

In the lymphoid compartment, TsEVs therapy led to a significant reduction in B and NK cell populations in the lungs (**Figure 6E**; **Supplementary Figure S3**). Although the proportion of CD4^+^ and CD8^+^ T cells remained unchanged, the increase in the total number of CD4^+^ T cells was observed (**Figure 6E**; **Supplementary Figures S3**, **S7B**). These findings demonstrate that the i.n. administration of TsEVs effectively mitigates the cellular and histopathological hallmarks of allergic airway inflammation.

### 3.5 TsEVs-induced protection against allergic airway inflammation is associated with the modulation of cytokine expression and the induction of Treg populations in the lung

**Figure 7:**
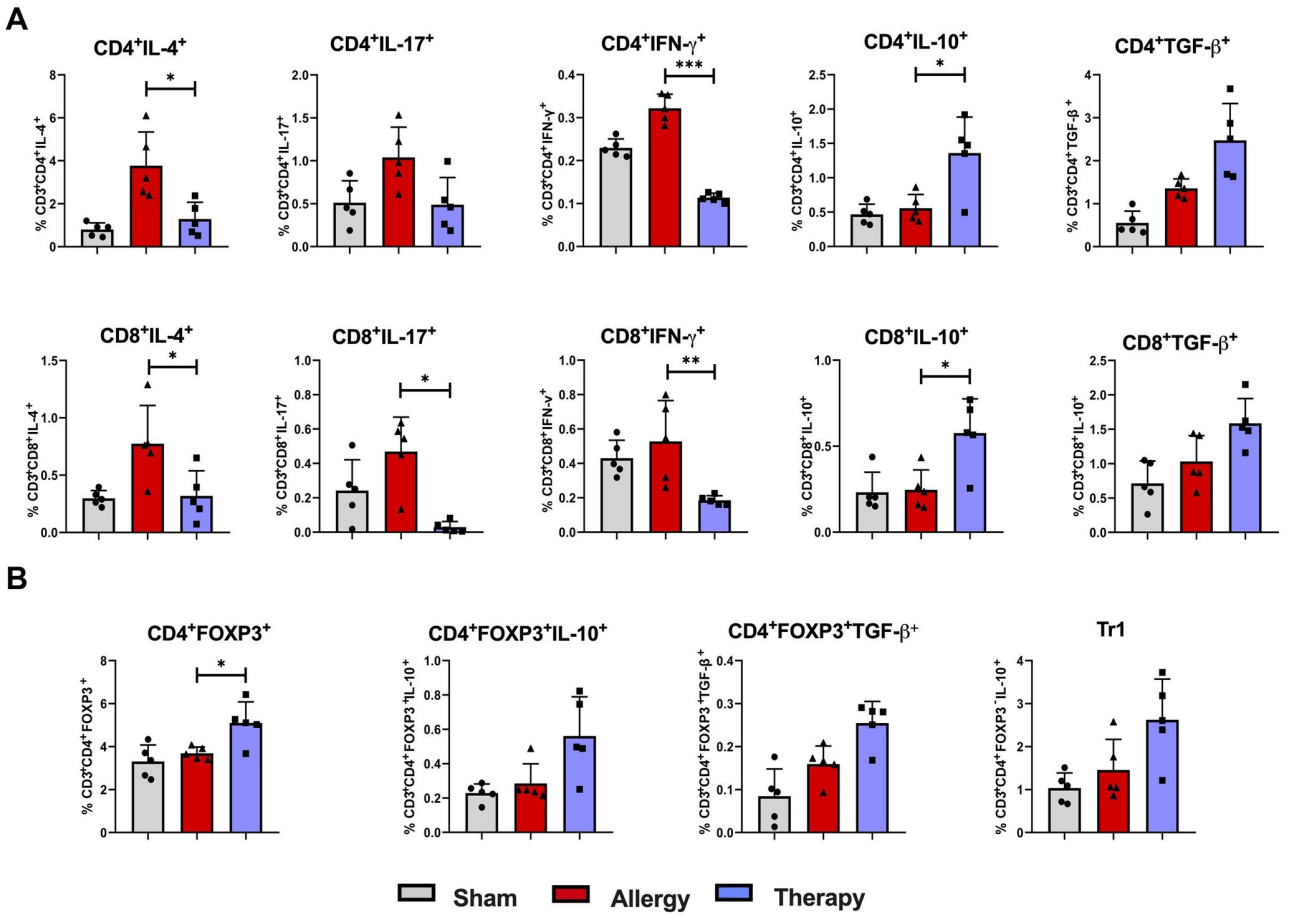
Cytokine expression and regulatory T cell responses in the lung following TsEVs treatments. Mice were treated as shown in the **Figure 2B**. Animals were sensitized with i.p. injections of ovalbumin and alum (OVA, 100 μg) on days 1 and 14 and challenged i.n. with OVA on days 22–24 (Allergy). The control group (Sham) received PBS instead of OVA. A treatment group received TsEVs (0.5 × 10^8^) via i.n. administration on days 19–21, and 30 minutes prior to each OVA challenge (days 22–24). On day 26 lung cells were collected and lymphoid cell subsets were analyzed using flow cytometry. Bar graphs display the percentage of cell subsets present in the lung. **(A)** The proportion of IL-4, IL-17, IFN-γ, IL-10, and TGF-β positive CD4^+^ and CD8^+^ T cells (see **Supplementary Figure S2** for gating strategy). **(B)** The proportion of regulatory T cell populations: Treg (CD4^+^Foxp3^+^) and Tr1 (CD4^+^Foxp3^-^IL-10^+^) cells and IL-10 and TGF-β positive Treg cells are presented (see **Supplementary Figure S3** for gating strategy). Differences were analyzed for significance by one-way ANOVA with Tukey’s posttest indicated as *p < 0.05, **p < 0.01 when compared with the Allergy group.

Analysis of cytokine expression in CD4^+^ lymphocytes revealed decreased levels of IL-4 and IFN-γ, alongside increased IL-10 expression, in TsEVs-treated groups compared to the Allergy group (**Figure 7A**; **Supplementary Figures S3**, **S7C**). Similarly, in the CD8^+^ lymphocyte compartment, TsEVs treatment led to reductions in IL-4, IL-17, and IFN-γ expression, coupled with elevated levels of IL-10 (**Figure 7A**). Regulatory T cell populations were evaluated based on Foxp3 expression (**Figure 7B**; **Supplementary Figures S4**, **S7D**). TsEVs-treated mice displayed a significant increase in the percentage and the total number of CD4^+^Foxp3^+^ Tregs in the lungs (**Figure 7B**). However, while the production of TGF-β by these cells was not enhanced in a statistically relevant manner, by TsEVs treatment managed to increase the total number of CD4^+^Foxp3^+^IL-10^+^ cells in the lungs (**Supplementary Figure S7D**). The proportions of Tr1 cells remained unchanged following TsEVs administration (**Figure 7B**).

### 3.6 Therapy with TsEVs modulates local and systemic recall responses to OVA *ex vivo*

**Figure 8:**
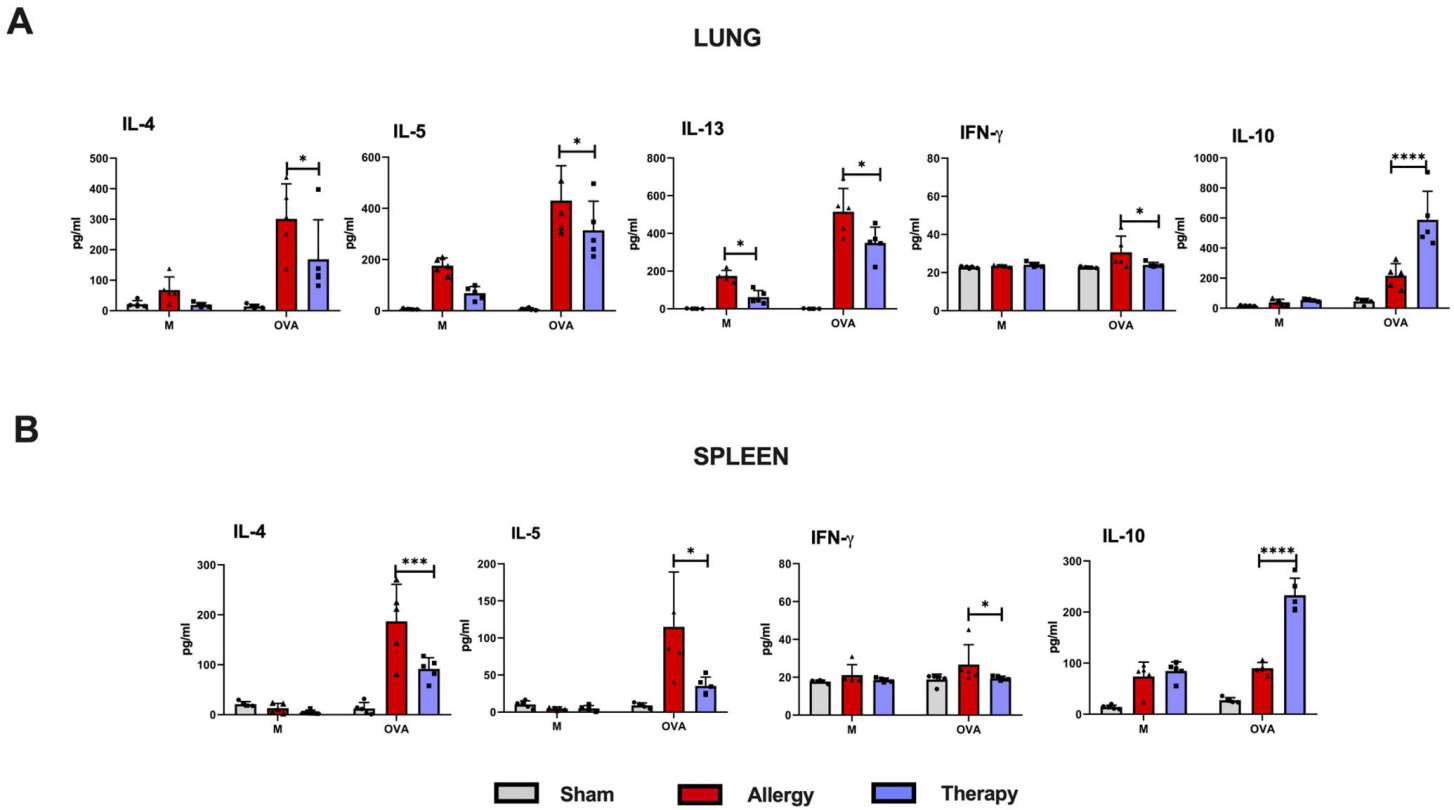
Recall responses in lung and spleen immune cells of TsEVs-treated or control mice. Mice were treated as shown in the **Figure 2B**. Lung **(A)** and spleen **(B)** cells were isolated from all examine groups (Sham, Allergy and Therapy group) and restimulated either with medium (M) or OVA (100 mg/ml). Supernatants were collected and cytokine levels were measured using ELISA. Data are presented as mean ± SD from three independent experiments. Statistical significance was determined using Two-way ANOVA with Tuckey’s multiple comparison test. *p < 0.05, ***p < 0.005, ****p < 0.0001 compared with the Allergy group.

To assess the effect of i.n. treatment with TsEVs on local and systemic immune responses, lung and spleen cells were isolated from Sham, Therapy and Allergy groups (**Figure 8**). These cells were restimulated with cognate allergen OVA, and cytokine levels were measured in the supernatants.

In the lung cell cultures, i.n. TsEVs treatment caused significant reduction in the production of pro-inflammatory cytokines IL-4, IL-5, IL-13, and IFN-γ compared to the Allergy group, while the level of the anti-inflammatory cytokine IL-10 was significantly increased (**Figure 8A**). Similarly, analysis of spleen cell cultures revealed that TsEVs-treated mice exhibited significant reduction in the levels of the Th2 cytokines IL-4 and IL-5, as well as the Th1 cytokine IFN-γ, following restimulation with OVA, compared to the Allergy group (**Figure 8B**). In contrast, TsEVs i.n. administration significantly increased the production of IL-10 (**Figure 8B**). These findings demonstrate that i.n. administration of TsEVs modulate both local and systemic immune responses, promoting an anti-inflammatory profile that likely underlies its protective effect against allergic airway inflammation.

### 3.7 TsEVs treatment reduces OVA-specific antibody levels in serum

**Figure 9:**
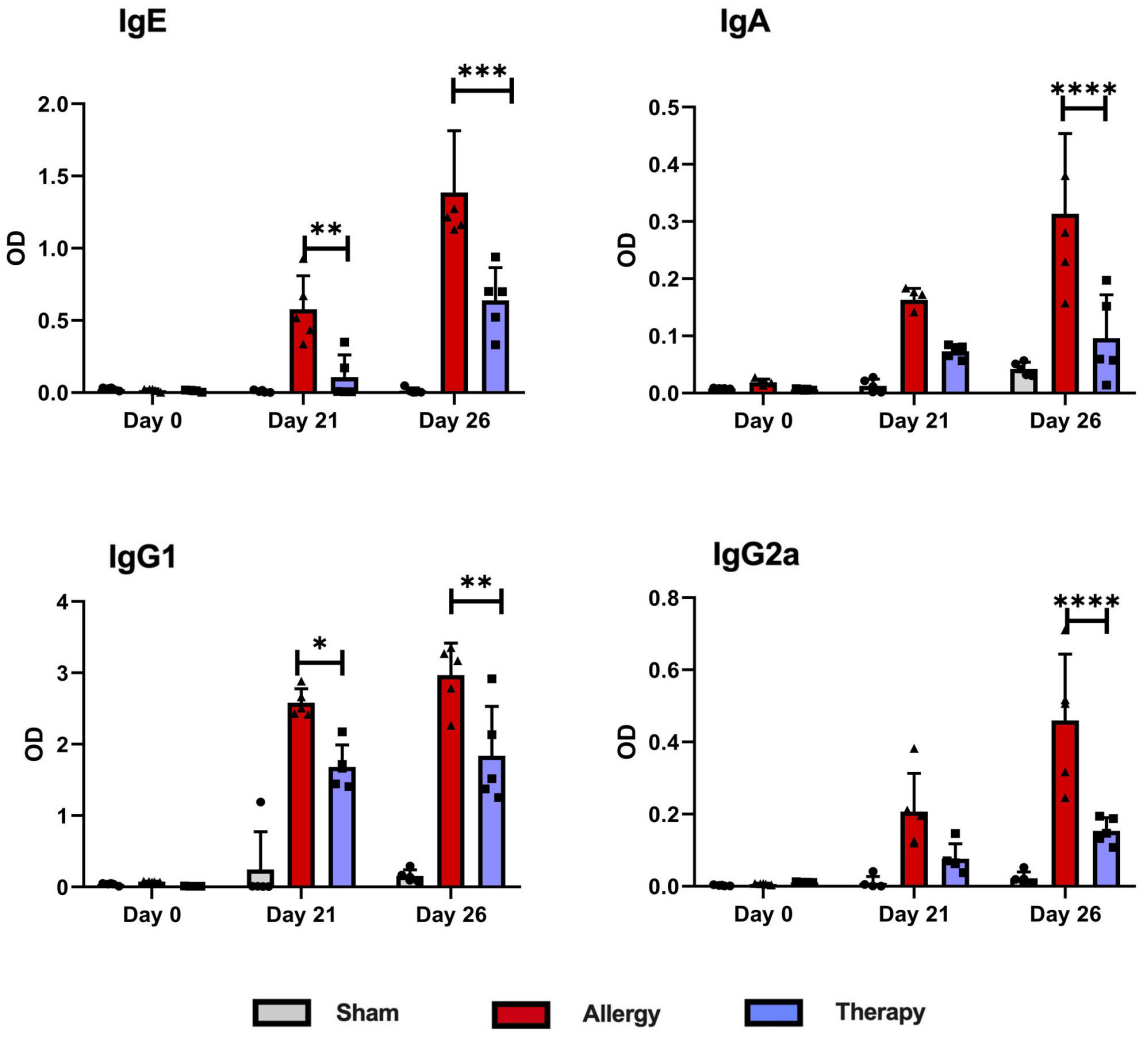
Levels of OVA-specific antibodies in serum following TsEVs treatments. Serum samples were collected from Allergy, Therapy and Sham group in three time points to measure the levels of OVA-specific IgE, IgA, IgG1 and IgG2a antibodies. Data are represented as mean ± SD from three independent experiments. Differences were analyzed for significance by Two-way ANOVA with Tuckey’s posttest indicated as *p < 0.05, **p < 0.01, ***p < 0.005, ****p < 0.0001 when compared with the Allergy group.

The effect of TsEVs treatment on systemic responses to allergen sensitization and challenge was evaluated by measuring OVA-specific antibody levels in mouse serum collected at three time points: before sensitization (day 0), prior to intranasal OVA challenge (day 21), and at the time of sacrifice (day 26) (**Figure 9**). Mice in the Allergy group exhibited significantly elevated levels of OVA-specific IgE, IgA, IgG1, and IgG2a antibodies throughout the experiment compared to the Sham group. TsEVs treatment effectively reduced these antibody levels relative to the Allergy group. IgE and IgG1 levels were significantly lower on day 21 and day 26, while IgA and IgG2 levels were significantly reduced only on day 26.

These findings demonstrate that although the TsEVs treatment was administered locally via i.n. delivery, its effects extended systemically. This systemic impact is evident by the significant modulation of OVA-specific antibody levels in serum and the observed alterations in cytokine production in splenocyte cultures. The ability of TsEVs to influence both local and distant immune responses highlights their potential as a therapeutic strategy for managing allergic airway inflammation.

### 3.8 *Ex vivo* treatment with TsEVs alters the cytokine profile of OVA-stimulated cells

**Figure 10:**
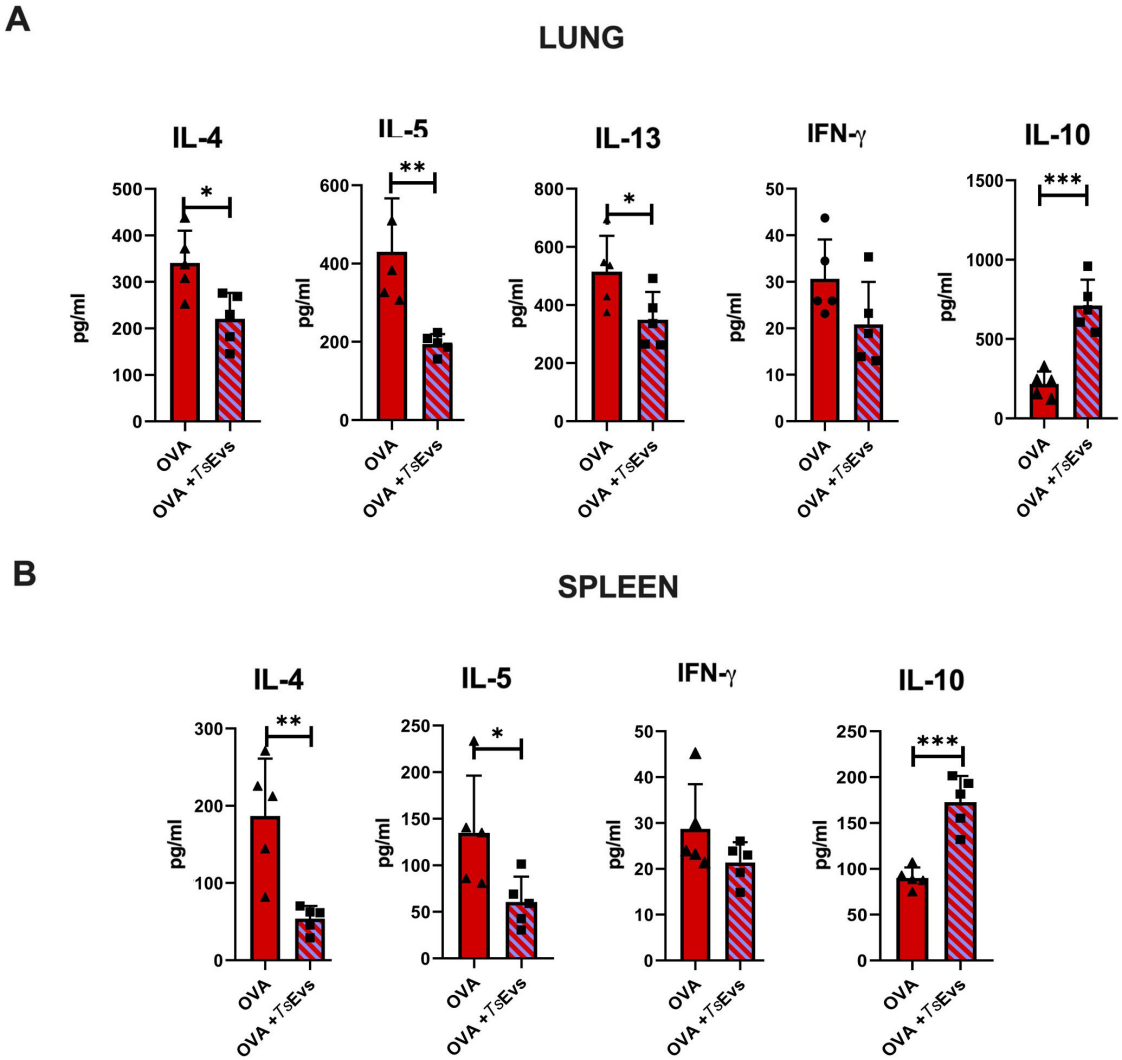
*Ex vivo* treatment of OVA-restimulated cells with TsEVs. Lung and spleen cells from the Allergy group were isolated and seeded for analysis. **(A)** Lung cells from the Allergy group were stimulated with OVA for 1h and subsequently treated with TsEVs (3x10^7^ particles/ml) for 72h **(B)** Spleen cells from the Allergy group were stimulated with OVA for 1h and then further treated with TsEVs for 72h. Cytokine levels in culture supernatants were measured by ELISA. Data are represented as mean ± SD from three independent experiments. Differences between OVA- and OVA+TsEVs - treated lung and spleen cells were analyzed using Student´s t-test with statistical significance represented by *p < 0.05, **p < 0.01, ***p < 0.005.

We also investigated the direct immunomodulatory potential of TsEVs on already activated immune cells. For this purpose, lung and spleen cells were isolated from the Allergy group, restimulated with OVA, and then treated either with TsEVs or with the medium alone (**Figure 10**). Cytokine levels were measured in the supernatants. In lung cell cultures, OVA-restimulated cells subsequently treated with TsEVs exhibited significant reduction in IL-4, IL-5 and IL-13 production, alongside elevated production of IL-10, compared to OVA-restimulated cells treated with medium (**Figure 10A**). TsEVs did not affect the production of IFN-γ. Similarly, spleen cell cultures treated with TsEVs after OVA restimulation showed a significant reduction in IL-4 and IL-5 levels compared to medium-treated controls, while IL-10 production was enhanced. As observed in lung cells, IFN-γ production remained unchanged (**Figure 10B**). These results highlight the potential of TsEVs to modulate the phenotype of already sensitized immune cells and shift the immune response toward a more anti-inflammatory profile.

## 4 Discussion

The presented study highlights the therapeutic potential of TsEVs in mitigating allergic airway inflammation. Our results show that TsEVs suppress type 2 immune responses while promoting regulatory pathways, making them a promising tool for the treatment of respiratory allergies without the disadvantages of infection with the whole parasite.

Consistent with previous studies demonstrating the immunomodulatory effects of *T. spiralis* infections in allergic conditions (30, 54, 55), we observed that i.n. administration of TsEVs, given as a therapy in already sensitized mice, alleviated allergy inflammation in the lungs, demonstrated by the lower numbers of eosinophils and neutrophils, reduced histopathology score due to reduced inflammatory cell infiltrations, mitigated mucus production, and also lowered production of Th2 cytokines. TsEVs application modulated the systemic immune response as well, reflected in reduced levels of OVA-specific serum IgE and IgG1 and impaired production of IL-4 and IL-5 cytokines by OVA-specific spleen cells.

The observed beneficial effect of TsEVs treatment may be the result of triggering the regulatory components of the immune response. Our previous work has shown that *T. spiralis* infection or i.p. application of its ES L1 products increases regulatory cytokine production and promotes the expansion Treg populations (28, 29, 32). Here, we show that i.n. administration of TsEVs in healthy mice induced an increase in the proportion and the total number of CD4^+^IL-10^+^ and CD8^+^IL-10^+^ cells. Further, TsEVs treatment elevated the proportion and the number of regulatory T cells, both CD4^+^Foxp3^+^ Tregs and CD4^+^Foxp3^-^IL-10^+^ Tr1, in the lungs, while in the spleen only the proportion of CD4^+^Foxp3^+^ Tregs was increased. Tr1 cell expansion observed in the lungs, the site of antigen exposure, was consistent with the intranasal route of TsEV administration. Tr1 cells function primarily as local regulators by suppressing naïve and memory T cell responses through the production of IL-10 (56). Therefore, the finding that the percentage of Tr1cells does not change at the systemic level under the influence of TsEVs is consistent with their known tendency to act locally where specific antigen is present.

TsEVs treatment suppressed the production of Th2 cytokines, IL-4, IL-5, and IL-13, key cytokines involved in driving allergic responses (57). IL-4 cytokine plays a pivotal role in regulating B cells during allergic reactions, upregulating IL-4 receptor and increasing B cell sensitivity to IL-4 signaling (58). This feedback mechanism sustains allergen-specific IgE antibody production while promoting B cell survival and proliferation during allergic inflammation (59). Monitoring the production of cytokines by immune cells of the lungs of TsEVs treated mice revealed lower levels of IL-4 cytokine and lower percentage and the total number of CD4^+^IL-4^+^ and CD8^+^IL-4^+^ T cells, compared to Allergy group. This reduction likely disrupts the feedback loop associated with allergic inflammation. It aligns with the decreased presence of B cells in the lungs and the decline in OVA-specific IgE, IgG1, and IgA antibodies. On the other hand, the differential timing in the reduction of antibody isotypes likely reflects the distinct kinetics and regulation of B cell responses (60). IgE and IgG1, which are closely linked to Th2-mediated responses and IL-4 signaling, appear more rapidly suppressed following TsEV-induced regulatory modulation. In contrast, IgA and IgG2a, which are associated with mucosal immunity and Th1-type responses, may require more sustained exposure to regulatory mechanisms for significant downregulation. It was reported that plasma cells producing different classes of immunoglobulins react differently to cytokines and other environmental signals (61).

Treatment with TsEVs additionally affected IL-5 cytokine production both in lung and in spleen cells. Cytokine IL-5 is an important player in the pathogenesis of allergic diseases since this cytokine promotes eosinophilic maturation, survival and eosinophilic inflammation (62, 63). Eosinophils release a variety of inflammatory mediators, including cytokines IL-4, IL-5, and IL-13, lipid mediators, and cytotoxic granule proteins. These mediators contribute to the inflammatory response associated with allergic reactions, promoting symptoms such as bronchoconstriction and mucus hypersecretion (64, 65). The decreased level of IL-5 in lung cells from mice treated with TsEVs is aligned with the reduction of the presence of eosinophils in BALF and lungs. Other cells that contribute to cytokine production in allergic reactions besides eosinophils are type 2 effector T cells, mast cells, type 2 innate lymphoid cells, and NK cells. NK cells proliferate in the presence of IL-4 and can contribute to the production of IL-4, IL-5, and IL-13 cytokines in an allergic reaction (66, 67). In line with this, the allergy group exhibited a significantly higher percentage of NK cells in the lungs, while therapy with TsEVs reduced their presence.

Particularly of note is the lower production of the IL-13 cytokine in the lungs of allergic mice treated with TsEVs. This cytokine plays a central role in the allergic reaction by regulating eosinophilia, mucus hypersecretion, fibrosis, and airway remodeling (68), but it should be noted that its increased production also accompanies parasitic infections. Studies in which parasite infection was induced to combat allergic inflammation reported that parasite infection did increase IL-13 cytokine production (31). However, this effect was not observed in our study, demonstrating that the use of TsEVs did not affect the immune response in the same way that parasite infection would.

The suppression of Th2 cytokine production observed in this study was not the result of Th1 cytokine release, since the expression of IFN-γ was reduced in both CD4^+^ and CD8^+^ T cells. Analysis of CD8^+^ T cells revealed that mice treated with TsEVs had reduced percentage of CD8^+^IL-17^+^ cells compared to the Allergy group. These cells have been linked to the pathology that occurs in autoimmune or allergic inflammatory diseases (69, 70), so it is possible that TsEVs alleviate allergy symptoms utilizing this mechanism as well.

During helminth infections, parasite evades the host immune response by releasing immunomodulatory antigens that facilitate an increase in the production of regulatory cytokines IL-10 and TGF-β (71, 72) by alternatively activated macrophages and tolerogenic DCs, which in turn drive Tregs development and expansion. Treg cells further release these cytokines, thereby maintaining the anti-inflammatory environment (73). We have previously shown that human DCs treated with TsEVs *in vitro* acquire a tolerogenic phenotype, characterized by increased secretion of IL-10 and TGF-β) and that such TsEVs-treated DCs possess the capacity to induce Tregs (47). IL-10 suppresses type 2 T cell immune response by downregulating IL-4-induced IgE production by B cells (74) or by inhibiting survival and activation of eosinophils and mast cells (75), while TGF-β can inhibit activation and proliferation of effector T cells (76). These cytokines also further stimulate the development of tolerogenic DCs and increase the presence of Treg cells.

The results of our study showed that TsEVs application in healthy mice stimulated the expression of IL-10 and TGF-β in CD4^+^ and CD8^+^ T cells, as well as the expansion of Treg cells, in both the lungs and spleen, and these effects were maintained in mice that were i.n. treated with TsEVs as a therapy for allergy. The fact that Treg subsets were not significantly increased in mice with established allergy that received TsEVs, may suggest that higher doses or prolonged administration of TsEVs are needed to induce their expansion under inflammatory conditions.

*Ex vivo* analysis provided additional support for the immunosuppressive potential of TsEVs. Treatment of OVA-restimulated lung and spleen cells from allergic mice with TsEVs resulted in reduced Th2 cytokine production and increased IL-10 expression. These results confirm the ability of TsEVs to modulate already-sensitized immune cells, which is critical for their therapeutic application. The production of IFN-γ, however, was not affected by TsEVs treatment, which is in contrast to the finding of *in vivo* experiment, where it was observed that the production of IFN-γ decreases following TsEVs administration. This discrepancy likely reflects the limited duration and simplified conditions of *ex vivo* exposure, which do not replicate the prolonged and repeated *in vivo* treatment.

## 5 Conclusions

Our findings demonstrate that TsEVs possess considerable therapeutic potential by promoting immune regulation and suppressing allergic inflammation without inducing adverse immune responses. The immunological mechanisms revealed here, positions TsEVs as a promising and innovative strategy for managing respiratory allergies and lays a foundation for designing safe helminth-based therapeutics. In this regard, the limitation of this study relates to the duration of TsEVs application, both in terms of sustained efficacy and rare adverse events or of-site effects. Also, efficacy of TsEVs should be tested in comparison to other treatment strategies, although TsEVs have an advantage of being allergen-independent option. However, the development of TsEVs-based therapeutics requires elucidation of the molecular mechanism underlying TsEVs´ immunomodulatory effects, especially the identification of a core set of bioactive molecules responsible for their function, which is the part of our ongoing and future efforts. Such knowledge would enable the design of synthetic vesicles or biomimetic formulations that replicate the beneficial properties of harnessing nature-inspired mechanisms, as exemplified by TsEVs, and may lead to the development of scalable, high-quality, and safe biologics for the treatment of allergy or other immune-mediated diseases.

## Data Availability

The raw data supporting the conclusions of this article will be made available by the authors, without undue reservation.
