# Supplementary material for: Trichinella spiralis-derived extracellular vesicles induce regulatory T cells and reduce airway allergy in mice
Source: Front Immunol. 2025 Jul 11;16:1637569. doi: 10.3389/fimmu.2025.1637569 (PMC12289487; doi:10.3389/fimmu.2025.1637569)
Supplement: Supplementary file 1 [file DataSheet1.pdf]

Supplementary Material

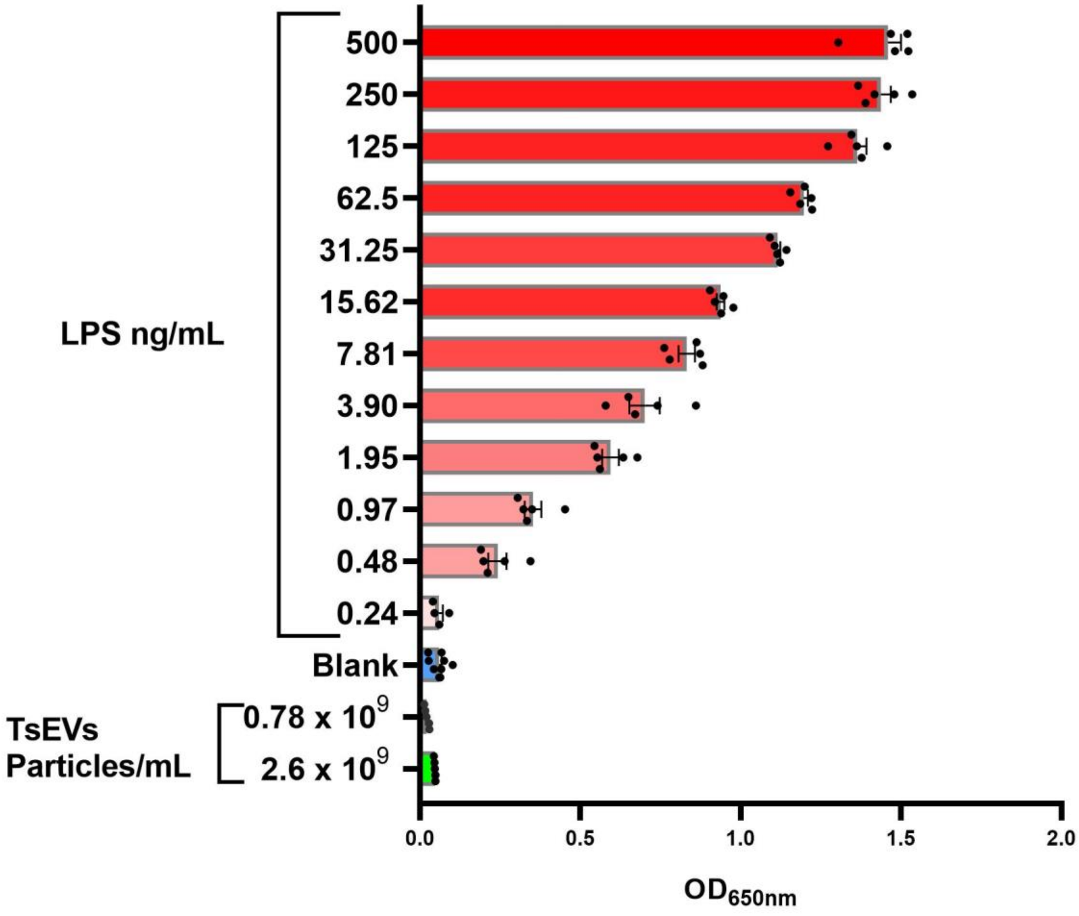

Supplementary figure S1. Detection of endotoxin in TsEVs. TsEVs were tested for presence of lipopolysaccharide (LPS) using HEK-Blue-mTLR4 cells HEK-Blue-mTLR4 cells carrying a SEAP reporter construct (InvivoGen, San Diego, CA, USA). The cells were maintained in high-glucose DMEM supplemented with 10% FCS, 2 mM L-glutamine, and 100 µg/mL Normocin, in the presence of the selection antibiotics (HEK-Blue™ Selection, Invitrogen). Cells were passaged upon reaching 70% confluence, following the manufacturer's recommendations. For the reporter assay, 2×10^4 cells were seeded in a 96-well plate in 180 µL of HEK-Blue™ Detection Medium (Invivogen). Two-fold serial dilutions of the TLR4 ligand (ultrapure LPS from E. coli O111:B4, Invivogen) were prepared in a range of 500 ng/mL to 0.24 ng/mL, along with TsEVs at 1.56×10^7 and 5.2×10^7, in 20 µL of HEK-Blue™ Detection Medium (corresponding to 0.78×10^9 and 2.6×10^9 Particles/mL), and added to the cells. The plate was incubated at 37°C with 5% CO_2 for the next 16 hours. SEAP activity was determined spectrophotometrically at 650 nm. Results are presented as mean OD ± SD from six replicates.

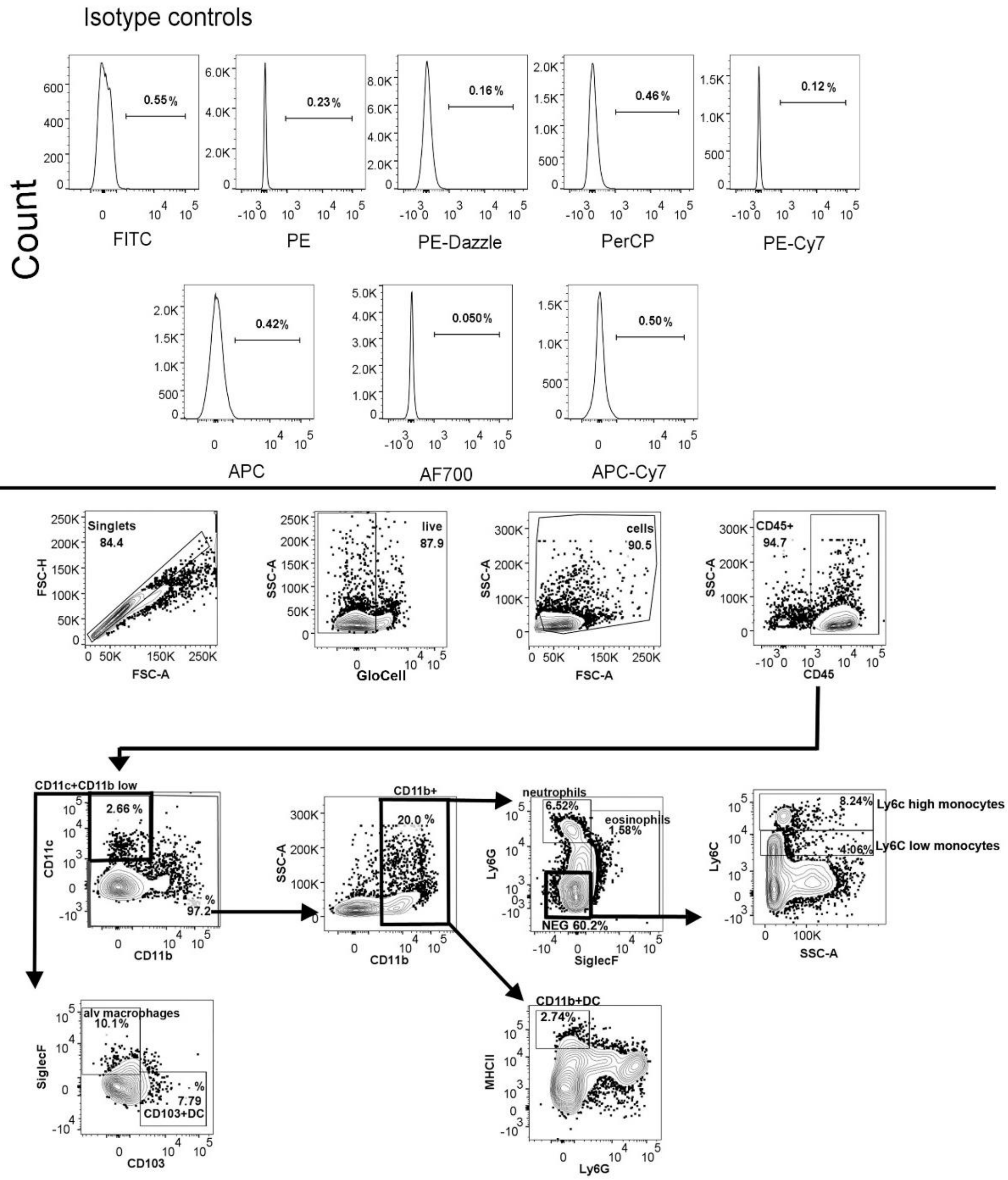

Supplementary Figure S2. Gating strategy for the myeloid cell subsets in the mouse lung. Cells were isolated from enzymatically digested mouse lungs, and after exclusion of doublets and dead cells (GloCell positive), immune cells were identified as CD45 postive. A sequential gating strategy was used to identify populations expressing CD11b and/or CD11c markers: withing CD11b^{+} cells, neutrophils (Ly6G^{+}SiglecF^{-}), eosinophyls (SiglecF^{+}) and Ly6C high or Ly6C low monocytes (Ly6G^{-}, SiglecF^{-}) were identified while CD11b^{+}DC were identified as MHC II (I-A)$^{+}$Ly6G^{-} cells. Within the CD11c^{+}CD11b low cell subpopulations, alveolar macrophages (SiglecF^{+}CD103^{-}) and CD103^{+}DC (SiglecF^{-}CD103^{+}) were identified. All percentages were calculated to total CD45^{+} cells. The specific fluorescence was determined according to single stained samples and isotype control antibodies. FSC, forward scatter; MHC II, major histocompatibility complex class II; SSC, side scatter.

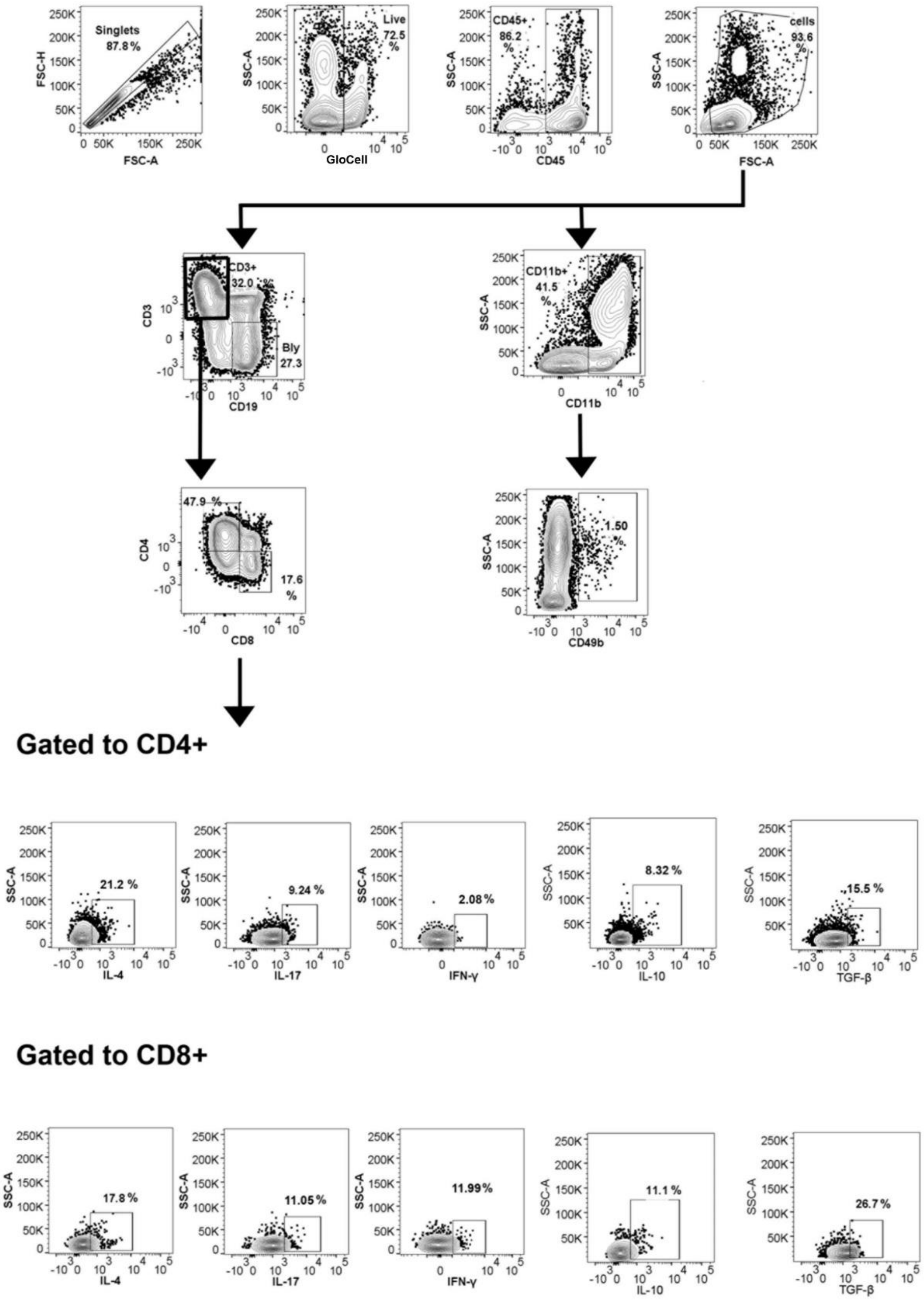

Supplementary Figure S3. Gating strategy for lymphoid cell subsets in the mouse lung. Lymphoid cell populations were identified by flow cytometry following exclusion of debris, doublets, dead cells (GloCell-positive), and non-immune cells (CD45^{-}). Live immune cells were gated as CD45^{+}. Natural killer (NK) cells were identified within the CD45^{+} population as CD11b^{+}CD49b^{+} cells and quantified relative to total CD11b^{+} cells. B lymphocytes were identified as CD3^{-}CD19^{+} within the CD45^{+} gate. T cells were defined as CD3^{+} and subsequently subdivided into CD4^{+} T helper and CD8^{+} cytotoxic T cell subsets. Intracellular cytokine-positive T cell populations were determined by further gating CD4^{+} or CD8^{+} cells based on expression of IL-4, IFN-γ, IL-17, IL-10, and TGF-β. All percentages were calculated to total CD45^{+} cells. A similar gating strategy was used to identify T cell subsets in the spleen.

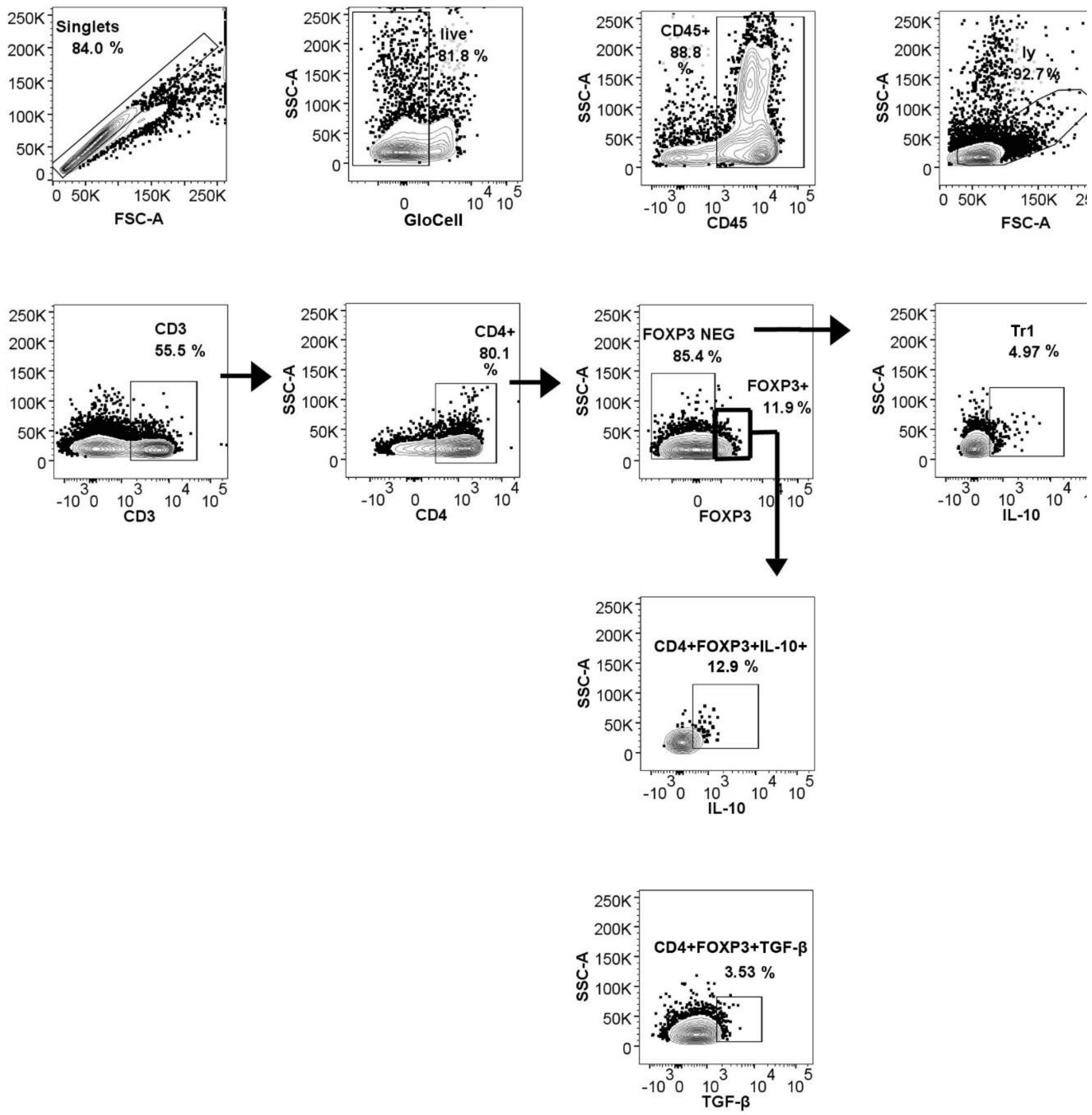

Supplementary Figure S4. Gating strategy for regulatory T cell analysis in mouse lungs. Lymphoid cell populations were identified by flow cytometry after excluding debris, doublets, dead cells (GloCell-positive), and non-immune cells ($CD45^{-}$). Live immune cells were gated as $CD45^{+}$, and T lymphocytes were identified within this population as $CD3^{+}CD4^{+}$ cells. Intracellular expression of IL-10 and TGF-β was then analyzed within the $CD3^{+}CD4^{+}$ subset. To assess regulatory T cell populations, $CD3^{+}CD4^{+}$ cells were further subdivided into two populations: $Foxp3^{+}$ cells, within which $IL\text{-}10^{+}$ and $TGF\text{-}\beta^{+}$ cells were analyzed, and $Foxp3^{-}IL\text{-}10^{+}$ cells, identified as type 1 regulatory T (Tr1) cells. All percentages were calculated to total $CD45^{+}$ cells. The same gating strategy was applied to analyze T lymphocyte subsets in the spleen.

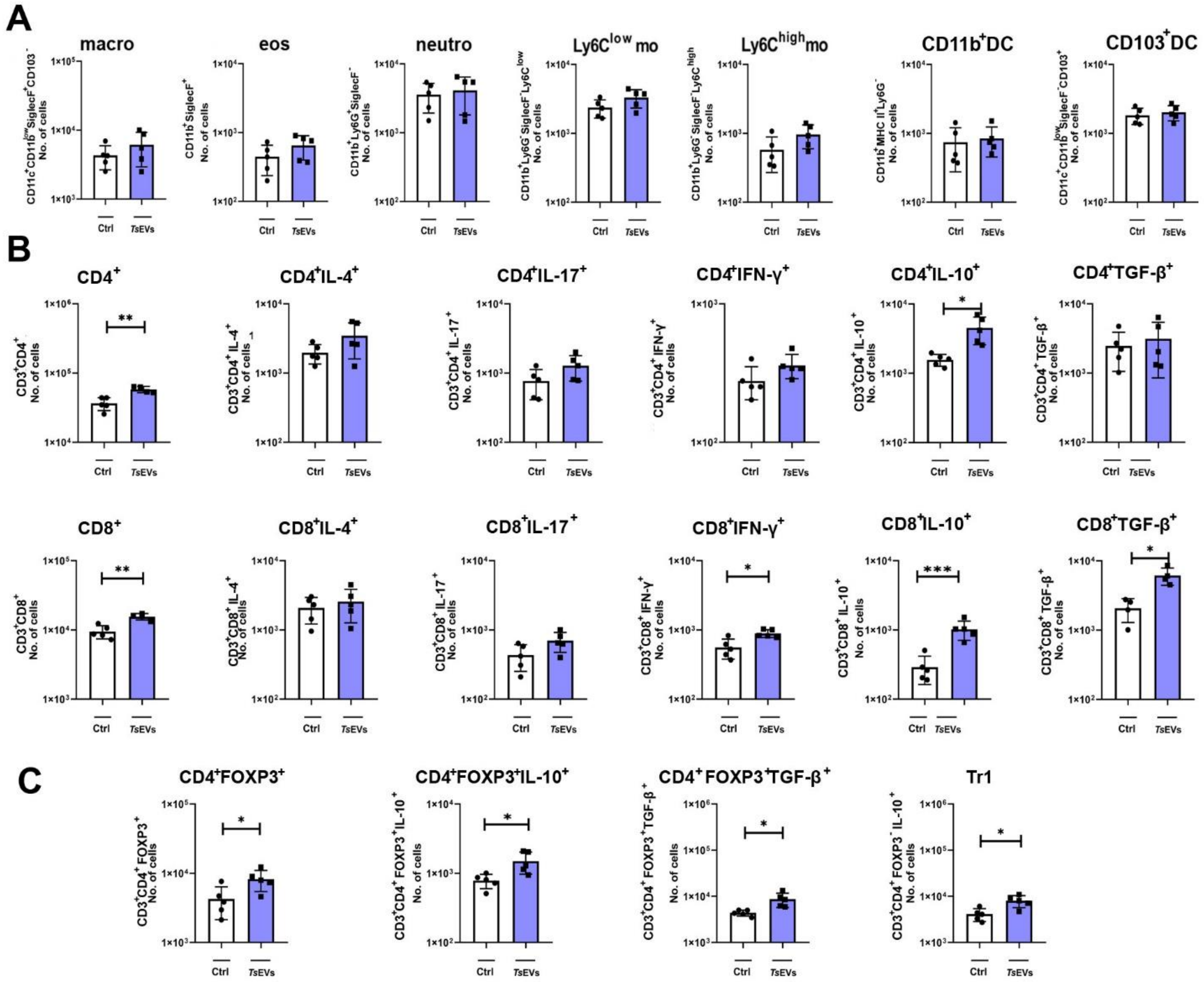

Supplementary Figure S5. Total cell numbers of myeloid and lymphoid cell populations in the lungs of mice treated with TsEVs. Mice were treated as outlined in **Figure 2A**: animals received either 0.5 × 10^8/30 µl TsEVs or equal volume of PBS (Ctrl) via intranasal administration daily for six consecutive days. Lung tissue was collected on day 8 and processed for flow cytometric analysis of immune cell populations. **(A)** Bar graphs display total number of myeloid cell subsets present in the lung which include macrophages (macro), eosinophils (eos), neutrophils (neutro), Ly6C^{low} and Ly6C^{high} monocytes (mo), CD11b$^+$ dendritic cells (CD11b$^+$ DC), and CD103$^+$ DC. Cell subset identification was based on established gating strategies shown in Supplementary Figure S1. **(B)** The total number of CD4$^+$ T cells and CD8$^+$ T cells, including subsets positive for intracellular staining of IL-4, IL-17, IFN-γ, IL-10, and TGF-β (see Supplementary Figure S2 for gating strategy). **(C)** The total number of CD4$^+$Foxp3$^+$ Tregs, CD4$^+$Foxp3$^-$IL-10$^+$ (Tr1) cells, and expression of IL-10 and TGF-β within CD4$^+$Foxp3$^+$ Treg cells (gating strategy shown in Supplementary Figure S3). Statistical comparisons were performed using Student's t-test and statistical significance indicated as $^*p < 0.05$, $^{**}p < 0.01$, $^{***}p < 0.005$.

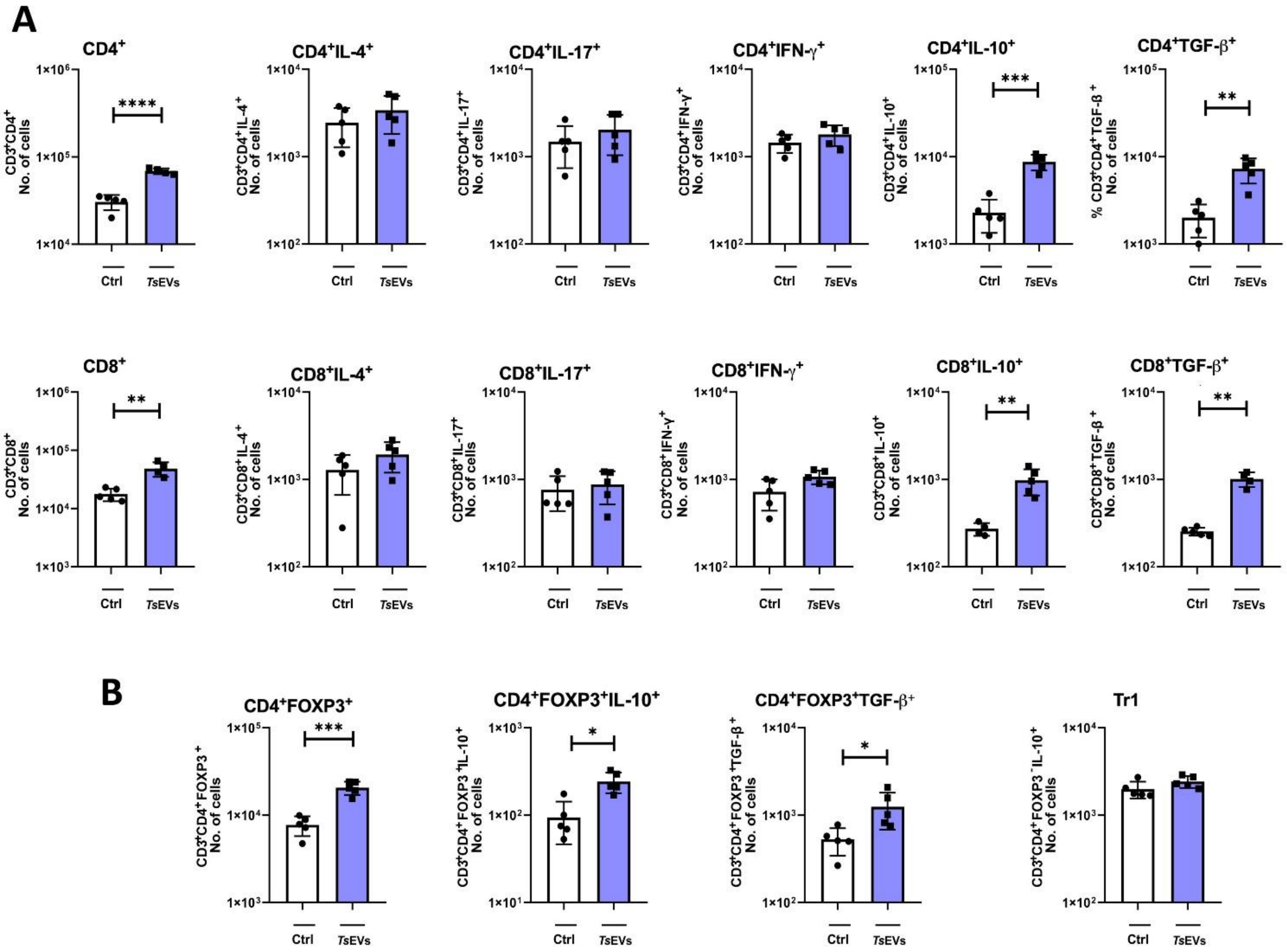

Supplementary Figure S6. Total cell numbers of lymphoid cell populations in the spleen of mice treated with TsEVs. Mice were treated as shown in the **Figure 2A.** Spleen cells were collected and analyzed using flow cytometry. **(A)** The total number of CD4$^+$ T cells, CD8$^+$ T cells, and IL-4, IL-17, IFN-γ, IL-10, TGF-β producing CD4$^+$ and CD8$^+$ T cells in spleen analyzed by flow cytometry (see Supplementary Figure S2 for gating strategy). **(B)** Total number of regulatory T cell populations: Treg (CD4$^+$Foxp3$^+$) and Tr1 (CD4$^+$Foxp3$^-$IL-10$^+$) cells and IL-10 and TGF-β producing Treg cells are presented (see Supplementary Figure S3 for gating strategy). *p < 0.05, **p < 0.01, ***p < 0.005, ****p < 0.0001 (Student´s t-test).

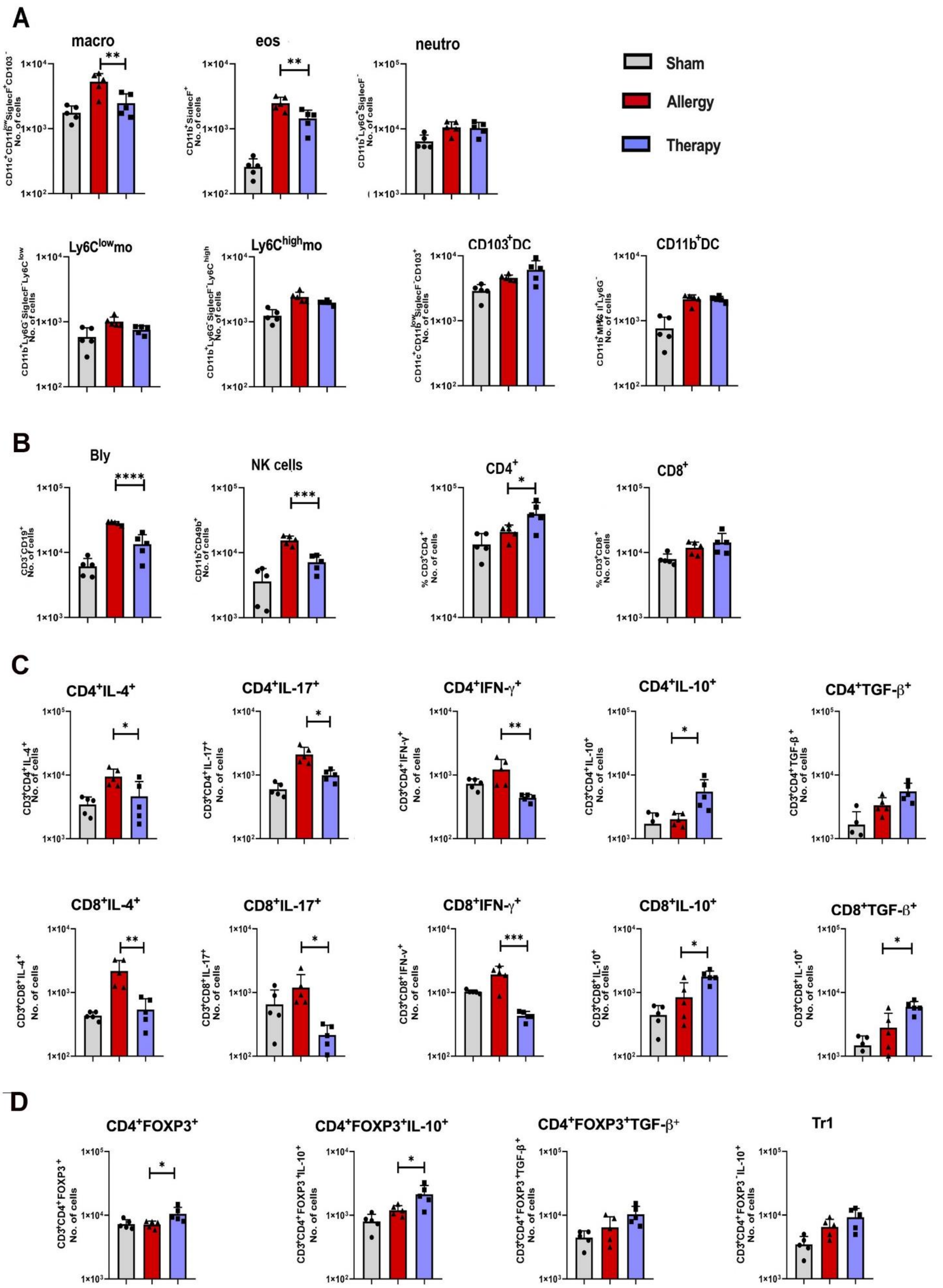

A
macro
eos
neutro
Sham
Allergy
Therapy
Ly6C^low mo
Ly6C^high mo
CD103+DC
CD11b+DC
B
Bly
NK cells
CD4+
CD8+
C
CD4+IL-4+
CD4+IL-17+
CD4+IFN-γ+
CD4+IL-10+
CD4+TGF-β+
CD8+IL-4+
CD8+IL-17+
CD8+IFN-γ+
CD8+IL-10+
CD8+TGF-β+
D
CD4+FOXP3+
CD4+FOXP3+IL-10+
CD4+FOXP3+TGF-β+
Tr1
No. of cells

Supplementary Figure S6. Total cell numbers of myeloid and lymphoid cell populations after TsEVs treatment in the lungs of mice with allergic inflammation. Mice were treated according to the protocol outlined in **Figure 2B**. Animals were sensitized with intraperitoneal (i.p.) injections of ovalbumin and alum (OVA, 100 µg) on days 1 and 14 and challenged with intranasal (i.n.) application of OVA on days 22–24 (Allergy). The control group (Sham) received PBS instead of OVA. The treatment group received TsEVs (0.5×10^8) via i.n. administration on days 19–21, and 30 minutes prior to each OVA challenge (days 22–24). **(A)** Bar graphs display the total number of myeloid cell subsets present in the lung: macrophages (macro), eosinophils (eos), neutrophils (neutro), $Ly6C^{low}$ and $Ly6C^{high}$ monocytes (mo), $CD103^+$ dendritic cells (DC) and CD11b+ DC (see Supplementary Figure S1 for gating strategy). **(B)** The total number of lymphoid cell subsets in the lung: B lymphocytes (Bly), natural killer (NK) cells, $CD4^+$ and $CD8^+$ T cells (see Supplementary Figure S2 for gating strategy). **(C)** The total number of IL-4, IL-17, IFN-γ, IL-10, and TGF-β positive $CD4^+$ and $CD8^+$ T cells (see Supplementary Figure S2 for gating strategy). **(D)** The total number of regulatory T cell populations: Treg ($CD4^+Foxp3^+$) and Tr1 ($CD4^+Foxp3^-IL\text{-}10^+$) cells and IL-10 and TGF-β positive Treg cells are presented (see Supplementary Figure S3 for gating strategy). Results are presented as mean ± SD of three independent experiments. Statistical analysis was performed using one-way ANOVA with Tukey's posttest and statistical significance indicated as *$p < 0.05$, **$p < 0.01$, ***$p < 0.005$, ****$p < 0.0001$ when compared with the Allergy group.
